# A Role for the Retinoblastoma Protein As a Regulator of Mouse Osteoblast Cell Adhesion: Implications for Osteogenesis and Osteosarcoma Formation

**DOI:** 10.1371/journal.pone.0013954

**Published:** 2010-11-11

**Authors:** Bernadette Sosa-García, Volkan Gunduz, Viviana Vázquez-Rivera, W. Douglas Cress, Gabriela Wright, Haikuo Bian, Philip W. Hinds, Pedro G. Santiago-Cardona

**Affiliations:** 1 Biochemistry Department, Ponce School of Medicine, Ponce, Puerto Rico; 2 Molecular Oncology Research Institute, Tufts Medical Center, Boston, Massachusetts, United States of America; 3 Molecular Oncology and Thoracic Oncology Departments, H. Lee Moffitt Cancer Center and Research Institute, Tampa, Florida, United States of America; University of Dayton, United States of America

## Abstract

The retinoblastoma protein (pRb) is a cell cycle regulator inactivated in most human cancers. Loss of pRb function results from mutations in the gene coding for pRb or for any of its upstream regulators. Although pRb is predominantly known as a cell cycle repressor, our data point to additional pRb functions in cell adhesion. Our data show that pRb regulates the expression of a wide repertoire of cell adhesion genes and regulates the assembly of the adherens junctions required for cell adhesion. We conducted our studies in osteoblasts, which depend on both pRb and on cell-to-cell contacts for their differentiation and function. We generated knockout mice in which the *RB* gene was excised specifically in osteoblasts using the *cre-lox P* system and found that osteoblasts from pRb knockout mice did not assemble adherens junction at their membranes. pRb depletion in wild type osteoblasts using RNAi also disrupted adherens junctions. Microarrays comparing pRb-expressing and pRb-deficient osteoblasts showed that pRb controls the expression of a number of cell adhesion genes, including cadherins. Furthermore, pRb knockout mice showed bone abnormalities consistent with osteoblast adhesion defects. We also found that pRb controls the function of merlin, a well-known regulator of adherens junction assembly, by repressing Rac1 and its effector Pak1. Using qRT-PCR, immunoblots, co-immunoprecipitation assays, and immunofluorescent labeling, we observed that pRb loss resulted in Rac1 and Pak1 overexpression concomitant with merlin inactivation by Pak1, merlin detachment from the membrane, and adherens junction loss. Our data support a pRb function in cell adhesion while elucidating the mechanism for this function. Our work suggests that in some tumor types pRb inactivation results in both a loss of cell cycle control that promotes initial tumor growth as well as in a loss of cell-to-cell contacts, which contributes to later stages of metastasis.

## Introduction

The retinoblastoma tumor suppressor protein (pRb) is a cell cycle repressor inactivated in most human cancers [1]–[5]. While the cell cycle regulatory pathway centered on pRb is inactivated in most human cancers [1], pRb itself is specifically inactivated with high frequency in a subset of human tumors, including retinoblastomas, osteosarcomas, and small cell lung carcinomas [4]. pRb can also be indirectly inactivated in other tumor types as a consequence of alterations targeting genes coding for any of its several upstream regulators such as CDK4, cyclin D and p16ink4a [6]. Independently of the inactivation mechanism, a predominant trait of the loss of pRb function is an inability to exit the cell cycle [7]. Interestingly, studies conducted in retinoblastomas, osteosarcomas, and small cell lung carcinomas point to an additional role for pRb as a regulator of cell adhesion. These tumor types show high frequencies of pRb inactivation and are composed of cells that lack stable adherens junctions, which are cadherin- and catenin-containing membrane complexes required for cell adhesion. In retinoblastomas, adherens junctions fail to anchor in the cortical actin cytoskeleton [8]. In osteosarcomas and small cell lung carcinomas, anomalous localization of adherens junction proteins has been observed, where cadherins and β-catenin show weak cytoplasmic expression [9], [10]. A strong correlation has been found in retinoblastomas and osteosarcomas between abnormal adherens junctions and invasive capacity [8], [9], underscoring the notion that disruption of adherens junctions-mediated cell adhesion is intimately related to metastasis.

The studies described above suggest that in some tumor types pRb inactivation results in both a loss of cell cycle control, which promotes initial tumor growth, as well as in a loss of cell-to-cell contacts, which later contributes to metastasis. This raises the possibility that pRb, in addition to its well-characterized role as a cell cycle repressor, may have a novel role as a regulator of cell-to-cell contacts and adherens junction formation. Remarkably, studies correlating pRb loss with adherens junction disruption have been largely unnoticed, and while pRb has been best characterized as a cell cycle regulator and its participation in developmental processes is still the subject of intense research, no molecular mechanism has been proposed to account for the correlation between pRb loss and adherens junction abnormalities.

We studied the link between pRb and adherens junctions within the context of osteoblast differentiation and bone formation, processes that depend on both pRb and on the establishment of cell-to-cell contacts [5], [11]–[14]. We generated conditional pRb knock-out mice in which the *RB* gene was excised specifically in osteoblasts using the cre-lox P system, followed by analyzes of the adhesive properties of osteoblasts obtained from these animals. In agreement with previous reports [8]–[10], we found that knocking out pRb production in osteoblasts had profound consequences on cell adhesion, altering the expression profile of osteoblast cadherins and other cell adhesion molecules, promoting disruption of adherens junctions, and producing abnormalities in bone structure. We found that pRb affects cell adhesion by at least two mechanisms. First, pRb controls the expression of cell adhesion genes. Second, pRb modulates the function of regulators of adherens junction assembly such as the small Rho GTPase Rac1 and the merlin tumor suppressor. Our data support a role for pRb as a regulator of adherens junction formation and cell adhesion and provide direct evidence that pRb depletion profoundly affects the capacity of cells to interact with one another.

## Results

### pRb-deficient osteoblasts do not undergo contact-dependent growth arrest

**Figure 1:**
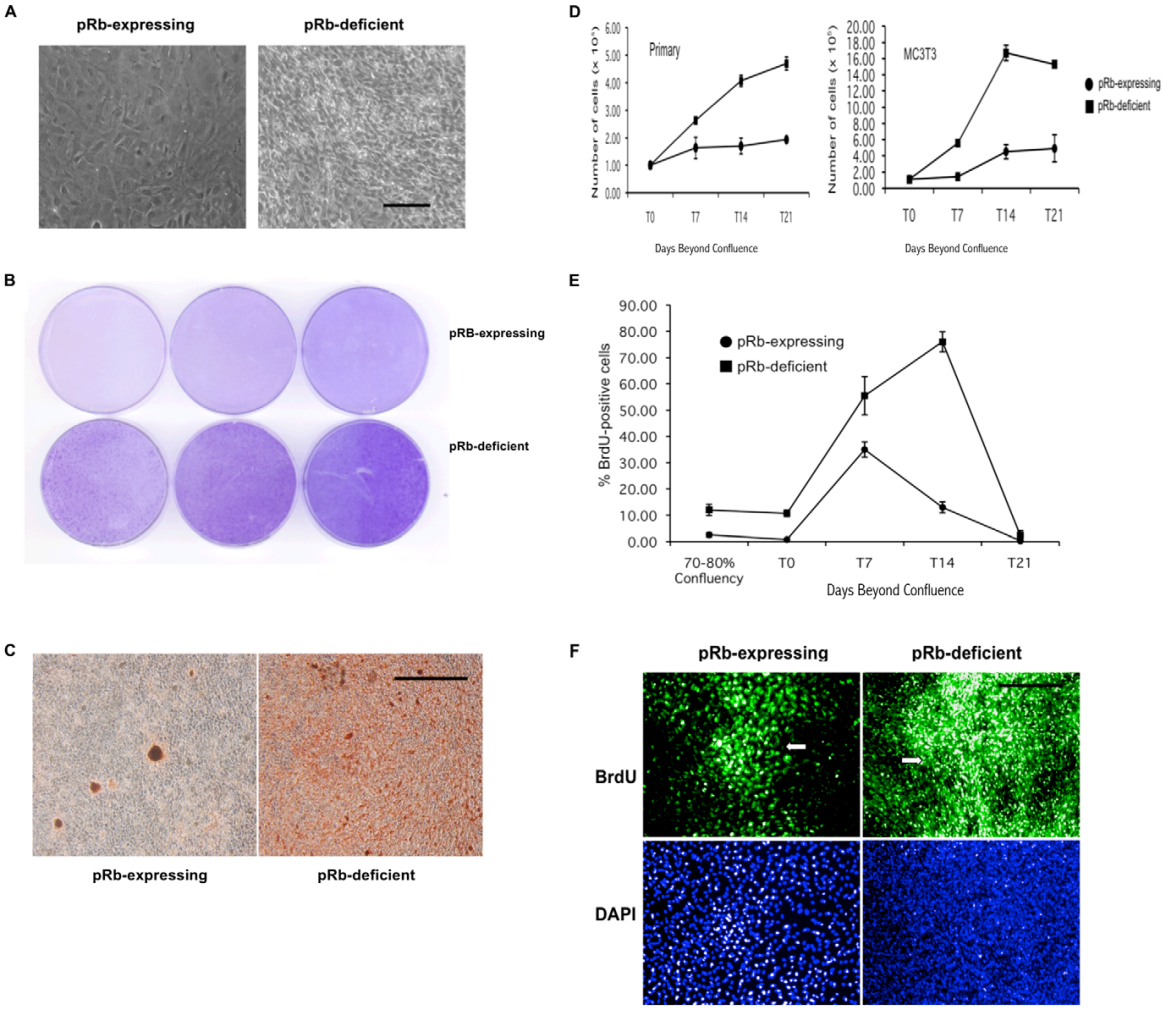
pRb-deficient osteoblasts do not undergo contact-dependent growth arrest. (**A**) Phase-contrast microscopy of osteoblasts cultured until 14 days beyond confluence (dbc). pRb-expressing osteoblasts form confluent monolayers (left) while pRb-deficient ones proliferate past confluence forming high-density cultures (right). Magnification = ×20. Bar = 20 µm. (**B**) Triplicates of MC3T3 cultures stained with crystal violet at 14 dbc, showing foci formed by pRb-deficient osteoblasts (bottom), compared to pRb-expressing osteoblasts (top). (**C**) Matrix mineralization in MC3T3 cultures. Phase contrast photomicrographs of alizarin red-S stained cultures at 10× showing increased extracellular matrix mineralization, indicative of increased density of bone-forming nodules, in pRb-deficient osteoblasts (right) relative to pRb-expressing controls (left). Magnification = ×10. Bar = 20 µm. (**D**) Growth curves showing that both pRb-deficient primary (left) and MC3T3 (right) osteoblasts grew to a saturation density ∼3.5-fold higher than their pRb-expressing counterparts. Each data point represents the mean of 3 independent experiments ± SEM. (**E**) BrdU immunocytochemistry of MC3T3 cells. Shown is percent of BrdU-positive cells, each data point representing the mean of 3 independent experiments ± SEM. Time points chosen for analysis were: before confluence (70–80%), confluence (T0), and 7, 14, and 21 dbc (T7, T14 and T21, respectively). (**F**) BrdU immunocytochemistry showing BrdU incorporation in MC3T3 cells at 7 dbc. While BrdU incorporation in pRb-expressing MC3T3 osteoblasts is restricted to bone forming nodules (arrow, top left), in pRb-deficient MC3T3 osteoblasts it is detected at both the nodules (arrow) and at their periphery (top right). Bottom panels show total nuclei in the same visual fields stained with DAPI. Magnification = ×10. Bar = 20 µm.

To study the effect of pRb loss in murine osteoblast cell adhesion, we produced primary cultures of these cells from animals lacking pRb as a result of targeted homologous recombination (pRb-deficient osteoblasts) as well as from their control littermates (pRb-expressing osteoblasts). Primary cultures were 3T3-immortalized (MC3T3) and used in subsequent experiments. Phase contrast microscopy of MC3T3 cultures showed that while pRb-expressing osteoblasts grow in culture to a confluent monolayer, pRb-deficient osteoblasts continue to proliferate past confluence, forming cell aggregates in which cells pile one on top of the other (Fig. 1A). These aggregates become apparent after 14 days in culture as crystal violet-stained foci (Fig. 1B). No only do pRb-deficient MC3T3 osteoblasts proliferate to higher densities, but they also have increased capacity to mineralize the extracellular matrix *in vitro*. This capacity depends in turn on the density of bone-forming nodules, which are active sites of bone mineral synthesis that arise by clonal expansion of a subpopulation of osteoblasts within a confluent monolayer. Staining of cultured osteoblasts with alizarin red-S, which stains bone mineral deposition, revealed increased mineralization by pRb-deficient osteoblasts relative to pRb-expressing controls (figure 1C), suggesting an increased density of bone-forming nodules in pRb-deficient cultures. This increased mineralization capacity has also been reported for primary pRb-deficient osteoblasts [15], demonstrating that pRb loss could be sufficient to alter osteoblast properties independently of additional mutations that drive immortalization or transformation.

In order to compare the growth properties of pRb-expressing and pRb-deficient osteoblasts in culture, primary and MC3T3 osteoblasts were plated at an initial density of 1×10^5^ cells per 6 cm culture plate and counted once per week for three weeks. This initial density produced confluent monolayers within 24 h after plating. While pRb-expressing osteoblasts showed little proliferation following initial plating, pRb-deficient primary and MC3T3 osteoblasts grew in culture to a density approximately 3.5-fold higher than their pRb-expressing counterparts (Fig. 1D).

To further test the proliferative behavior of these cells, MC3T3 osteoblasts were processed for BrdU immunocytochemistry before reaching confluence (70–80%), at confluence (T0), and at 7, 14, and 21 days beyond confluence (dbc). pRb-expressing and pRb-deficient MC3T3 osteoblasts showed similar kinetics of BrdU incorporation up until 7 dbc. After peaking at 7 dbc, pRb-expressing osteoblasts showed a significant reduction at 14 dbc, a time point at which pRb-deficient MC3T3 osteoblasts still showed robust BrdU incorporation. BrdU-immunoreactive cells were undetectable at 21 dbc in both pRb-deficient and pRb-expressing MC3T3 osteoblast cultures (Fig. 1E). As expected, BrdU incorporation in pRb-expressing MC3T3 osteoblasts at 7 dbc was restricted to cells undergoing clonal expansion within bone-forming nodules, while being rare at the periphery of the nodules (Fig. 1F, top left, arrow). In contrast, pRb-deficient MC3T3 cells showed robust BrdU incorporation in both the nodules and their periphery (Fig. 1F, top right, arrow indicates bone forming nodule). Together, these data show that pRb-deficient osteoblasts grow to high densities relative to pRb-expressing osteoblasts, resist contact-dependent growth arrest, and are poorly responsive to conditions of high cell density that led to proliferative arrest of their pRb-expressing counterparts. These observations suggest that they are defective in sensing cell-to-cell contacts with neighboring cells. pRb-deficient MC3T3 osteoblasts also showed other traits indicative of transformation, such as forming tumors when injected into mice and growing in an anchorage-independent manner (Fig. S1).

### pRb-deficient osteoblasts lack adherens junctions

**Figure 2:**
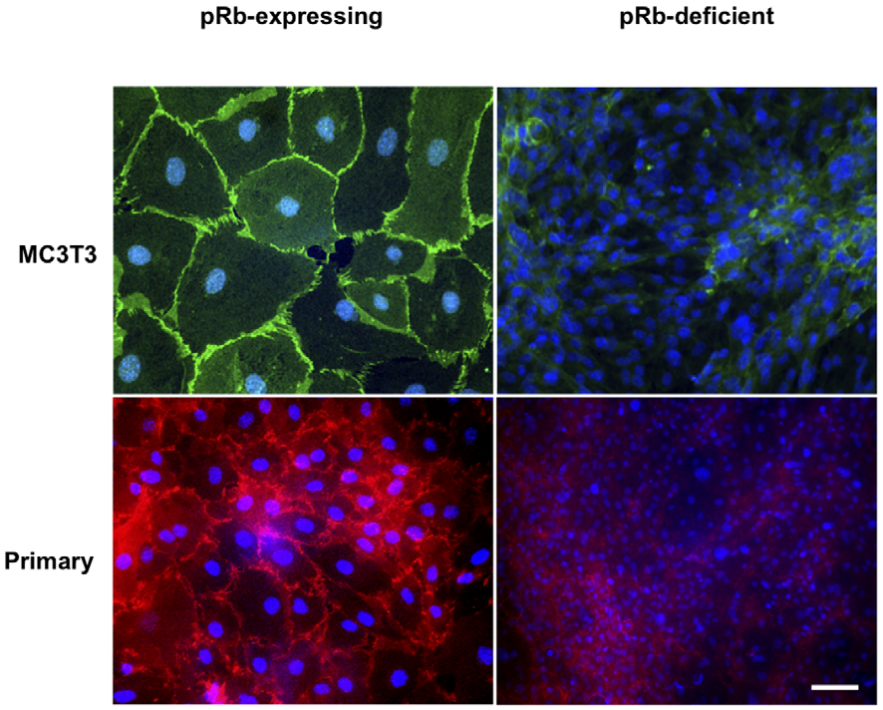
Abrogation of pRb expression disrupts adherens junctions. Immunocytochemical localization of β-catenin in MC3T3 (top) and primary osteoblasts (bottom). pRb-expressing osteoblasts show strong β-catenin membrane-associated labeling along areas of cell-to-cell contact (left) while pRb-deficient osteoblasts show a weak and diffuse labeling, without any clearly discernible membrane labeling (right). Magnification = ×100. Bar = 1 µm.

The resistance of pRb-deficient osteoblasts to undergo contact-dependent growth arrest could result from a failure to form adherens junctions. Therefore, we examined the status of these structures by immunocytochemical labeling of β-catenin, a predominant adherens junction protein. While pRb-expressing MC3T3 osteoblasts showed strong membrane-associated β-catenin labeling, suggesting intact adherens junctions (Fig. 2, top left), pRb-deficient MC3T3 osteoblasts showed a weak, diffuse cytoplasmic staining, without any discernible membrane labeling (Fig. 2, top right). The β-catenin staining observed in pRb-deficient osteoblasts is indistinguishable from that reported in osteosarcomas and small cell lung carcinomas [9], [10]. To rule out the possibility that the loss of adherens junctions observed in pRb-deficient MC3T3 osteoblasts could be an artifact of the immortalization process, we assessed the integrity of these structures in primary osteoblasts. Similar to their 3T3-immortalized counterparts, pRb-expressing primary osteoblasts showed intact adherens junctions (Fig. 2, bottom left), while these structures where absent in pRb-deficient primary osteoblasts (Fig. 2, bottom right). This demonstrates that pRb loss could be sufficient to alter osteoblast adhesive properties independently of additional mutations that drive immortalization or transformation. Knocking down endogenous pRb in pRb-expressing osteoblasts with *RB*-shRNA also resulted in adherens junction loss (Fig. S2). Interestingly, we also observed a decreased cell size in pRb-deficient osteoblasts relative to their pRb-expressing counterparts. This decreased size was observed in both primary osteoblasts and their 3T3-immortalized derivatives, although it was more notable in primary osteoblasts (figure 2, right panels). Although we currently lack a mechanistic explanation linking pRb loss to decreased cellular volume, we speculate that pRb loss could impinge upon checkpoints beyond the G1/S transition, specifically upon those that ensure that a cell does not progress to mitosis until it has achieved a certain volume.

### pRb-deficient osteoblasts show altered cadherin expression

**Figure 3:**
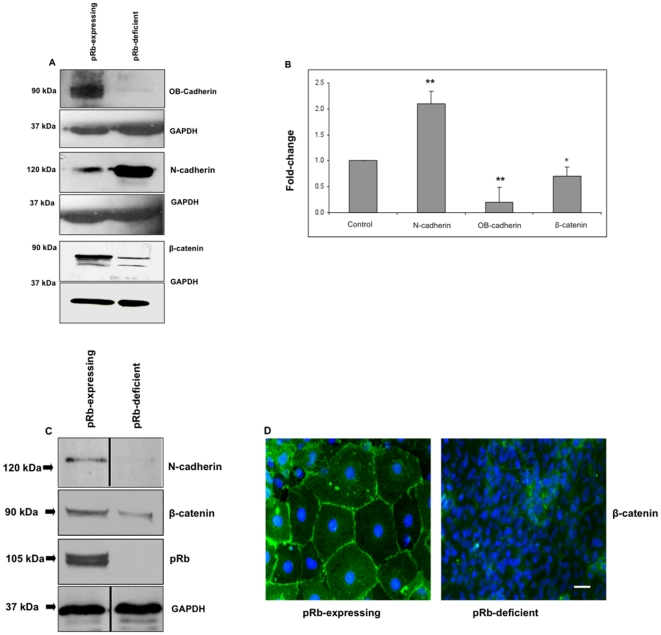
pRb deletion leads to abnormal cadherin expression. (**A**) Immunoblot of confluent cultures showing decreased levels of OB-cadherin (top panel) and β-catenin (bottom panel) and increased levels of N-cadherin (middle panel) in pRb-deficient cells relative to pRb-expressing controls. In the β-catenin panel, the top band corresponds to β-catenin while the lower bands are irrelevant background bands. Representative blots of at least 3 independent experiments are shown. Equal loading of the lanes is shown by GAPDH levels for each individual immunoblot. (**B**) qRT-PCR showing levels of OB-cadherin, N-cadherin, and β-catenin mRNAs in pRb-deficient osteoblasts relative to pRb-expressing osteoblasts (control bar). Each bar represents the average of at least 3 independent experiments ± SEM. Values were normalized against GAPDH. **, *P*<0.005; *, *P*<0.05. (**C**) Representative immunoblots of at least 3 independent experiments of osteoblasts cultured until 14 dbc showing that the initial up-regulation of N-cadherin levels observed in pRb-deficient osteoblasts is not sustained after prolonged culture time (top). β-Catenin expression is also reduced in pRb-deficient osteoblasts at 14 dbc (second from top). The pRb status of these cells was confirmed (third panel from top), and equal loading of the lanes is demonstrated by GAPDH levels (bottom). (**D**) Immunofluorescent labeling of β-catenin showing lack of adherens junctions in pRb-deficient osteoblasts cultured until 14 dbc (right), compared to pRb-expressing osteoblasts cultured in the same conditions (left). Magnification = ×100. Bar = 1 µm.

To determine if pRb loss affects the expression of osteoblast cadherins, we performed immunoblot analyses of MC3T3 osteoblasts cultured until confluence. We found that while pRb-expressing osteoblasts showed high OB-cadherin expression, pRb deletion dramatically reduced the levels of this cadherin (Fig. 3A, top panel). Intriguingly, pRb-deficient osteoblasts also showed elevated levels of N-cadherin relative to the basal levels observed in pRb-expressing controls (Fig. 3A, middle panel), suggesting a compensation for OB-cadherin loss by a proportional increase in N-cadherin. In agreement with our β-catenin immunofluorescence studies, immunoblots showed reduced β-catenin levels in pRb-deficient osteoblasts relative to pRb-expressing controls (Fig. 3A, bottom panel, the top band corresponds to β-catenin while lower bands represent irrelevant background bands). qRT-PCR analyses of MC3T3 osteoblasts cultured until confluence showed decreased OB-cadherin and β-catenin mRNA and increased N-cadherin mRNA levels in pRb-deficient osteoblasts relative to pRb-expressing controls (Fig. 3B).

The increase in N-cadherin expression observed in pRb-deficient osteoblasts is intriguing given the absence of adherens junctions in these cells. Therefore, we investigated if the elevated N-cadherin levels persist in pRb-null osteoblasts after prolonged time in culture. Whereas pRb-expressing osteoblasts sustained their basal N-cadherin expression at 14 dbc, N-cadherin expression was decreased in pRb-deficient osteoblasts at this time point (Fig. 3C, top panel). Furthermore, whereas pRb-expressing osteoblasts show defined adherens junctions at 14 dbc, prolonged culturing did not reestablish adherens junction formation in pRb-deficient osteoblasts, as evidenced by the low levels of β-catenin (Fig. 3C, second panel from top) and by their lack of membrane-associated β-catenin immunostaining (Fig. 3D). Thus, we speculate that in a pRb-null background N-cadherin becomes susceptible to degradation in the absence of adherens junctions to which it can attach. Finally, immunoblot analyses confirmed the pRb status of pRb-expressing and pRb-null osteoblasts (Fig. 3C, third panel from top). Together, our results show that pRb is required for the proper regulation of OB- and N-cadherin expression in osteoblasts and for their assembly into stable adherens junctions.

### pRb knockout mice show abnormal calvarial bones

**Figure 4:**
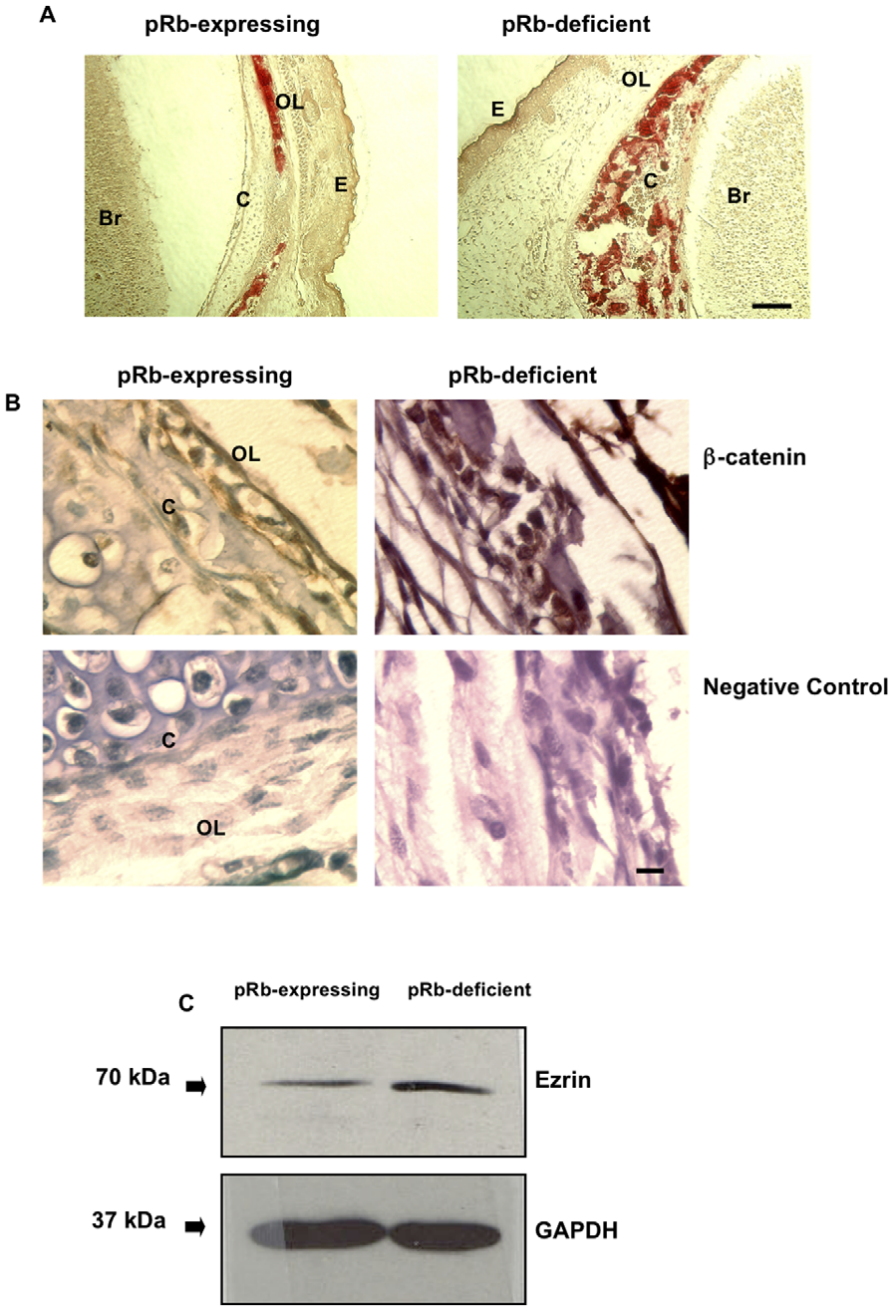
Osteoblasts in the calvarial bone of conditional pRb knockout mice show abnormal β-catenin staining. (**A**) Coronal skull sections of embryonic day 18.5 (E18.5) mice stained with alizarin red-S showing location of differentiated osteoblasts. In calvarial bones from pRb-expressing mice (left), osteoblasts form a layer lining the cartilage at the base of the skull, while in pRb knockout mice (right) scattered calcified nodules are found within a grossly disorganized cartilage. Magnification = ×4. Bar = 2 µm. (**B**) β-Catenin immunohistochemistry of calvarial bones of pRb-expressing and pRb knockout mice. Osteoblasts in calvarial bones from pRb-expressing mice show membrane β-catenin (top left). Clusters of cells showing strong nuclear immunoreactivity for β-catenin were found within the disorganized cartilage in pRb knockout mice (top right). Negative controls processed without primary antibody are shown (bottom). OL, osteoblast layer; E, epithelium; C, cartilage; Br, brain. Magnification = ×100. Bar = 1 µm. (**c**) A representative immunoblot showing increased ezrin expression in cultured pRb-deficient osteoblasts relative to pRb-expressing controls.

Next, we studied the calvarial bones in coronal sections of E18.5 pRb-null and control littermate skulls. To localize the calvarial bone, sections were stained with alizarin red-S to identify areas of active extracellular mineral deposition indicating the presence of differentiated osteoblasts. Calvarial bones from control mice displayed an osteoblast layer lining the cartilage at the base of the skull (Fig. 4A, left). In pRb knockout mice this cartilage is grossly disorganized and instead of a defined osteoblast layer, scattered calcified nodules are apparent in the space that would normally by occupied by cartilage (Fig. 4A, right). Immunohistochemistry revealed that in control mice the osteoblast layer consists of cells with membrane-associated β-catenin (Fig. 4B, top left). Interestingly, clusters of cells showing nuclear β-catenin immunoreactivity were found within the disorganized cartilage in pRb knockout mice (Fig. 4B, top right). pRb is thus required to properly anchor β-catenin to the cell membrane also *in vivo,* and the observed aberrant β-catenin localization due to pRb loss is not an artifact of culture conditions.

The phenotype of the calvaria of pRb knockout mice is consistent with pRb-deficient osteoblasts' defect in establishing cell-to-cell contacts, which could allow them to migrate away from their proper position in the calvaria and to invade the adjacent cartilage. Supporting this, pRb-deficient osteoblasts expressed elevated levels of ezrin (Fig. 4C), a membrane-cytoskeleton linker whose up-regulation is considered an indicator of osteosarcoma metastasis [16], [17].

### pRb loss results in unrestrained Rac1 activity and merlin inactivation

**Figure 5:**
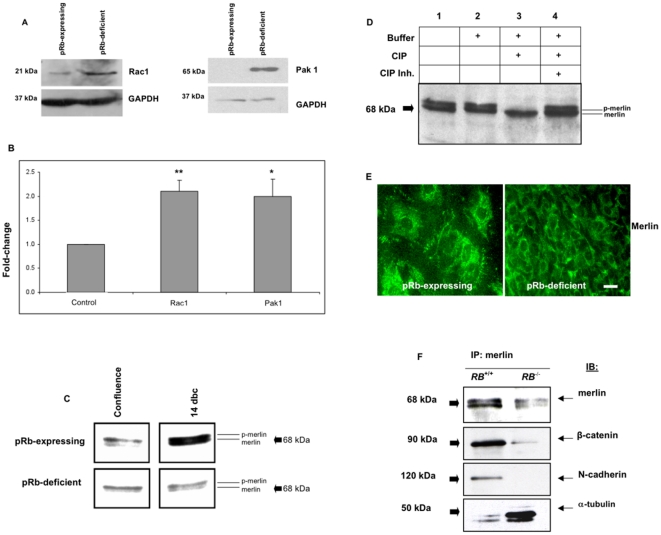
pRb loss results in unregulated Rac1, Pak1, and merlin function. (**A**) Immunoblot showing increased Rac1 (left) and Pak1 (right) levels in pRb-deficient osteoblasts relative to pRb-expressing controls. Equal loading of lanes is shown by GAPDH levels (bottom panel for each protein). (**B**) qRT-PCR showing the same upregulation at the mRNA level in pRb-deficient osteoblasts relative to pRb-expressing osteoblasts (control bar). Each bar represents the average of at least 3 independent experiments ± SEM. Values were normalized against GAPDH. **, *P*<0.005; *, *P*<0.05. (**C**) Immunoblot showing an increased ratio of the hypophosphorylated to the hyperphosphorylated merlin in pRb-expressing MC3T3 osteoblasts at 14 dbc. (**D**) Whole protein extracts from pRb-expressing MC3T3 osteoblasts at 14 dbc treated with calf intestinal phosphatase (CIP) before immunoblot analysis for merlin. CIP treatment eliminated the slower-migrating band, whereas CIP inhibitors reversed this effect. Lane 1, untreated lysates; lane 2, lysates treated only with CIP buffer; lane 3, lysates treated with CIP; lane 4, lysates treated with CIP plus inhibitors. (**E**) Immunocytochemical localization of merlin in MC3T3 cells. In pRb-expressing MC3T3 osteoblasts merlin staining reveals a strong, punctuated perinuclear pattern as well as a membrane staining (left). In pRb-deficient MC3T3 osteoblasts cells the membrane staining is lost (right). Magnification = ×100. Bar = 1 µm. (**F**) Merlin was immunoprecipitated from whole protein lysates followed by immunoblots of the immunoprecipitates with antibodies against merlin, β-catenin, N-cadherin, and α-tubulin. In pRb-expressing MC3T3 osteoblasts, merlin is immunoprecipitated predominantly as a hypophosphorylated form together with adherens junction components β-catenin and N-cadherin. In pRb-deficient MC3T3 osteoblasts, merlin is immunoprecipitated predominantly as a hyperphosphorylated form together with α-tubulin.

We examined the possibility that pRb could regulate cell adhesion by controlling adherens junctions assembly, thus we hypothesized that pRb controls the function of important regulators of this process. We focused our attention on the small Rho GTPase Rac1 and its effector, the p21-activated protein kinase 1 (Pak1), and the merlin tumor suppressor. We had a solid rationale for studying these molecules. First, Rac1 is a known regulator of adherens junction stability and its unrestrained activity disrupts adherens junctions [18]; second, pRb represses Rac1 in osteoblasts [19], [20]; third, merlin is a membrane protein required for adherens junction assembly and contact-dependent growth arrest [21]; and fourth, merlin is a Rac1 target that is inactivated upon phosphorylation at serine 518 (Ser518) by Rac1s effector Pak1 [22]–[24]. Therefore, we postulated that pRb represses Rac1 and Pak1 expression, thus allowing merlin activity and adherens junction assembly at the cell membrane. We tested this by assessing Rac1 and Pak1 expression with immunoblots and qRT-PCR. Supporting our hypothesis, levels of Rac1 and Pak1 proteins and mRNA were increased in pRb-deficient osteoblasts compared with pRb-expressing controls (Figs. 5A and B).

Next, we compared pRb-expressing with pRb-deficient MC3T3 osteoblasts in terms of merlin function. Immunoblots showed that merlin migrates as a doublet (Fig. 5C), consistent with it being a phosphoprotein existing in hyper- and hypophosphorylated forms [22]–[24]. At the time of confluence, merlin was detected predominantly as the slower-migrating hyperphosphorylated form in both pRb-expressing and pRb-deficient MC3T3 cultures (Fig. 5C, left panels). However, we observed an increase in the ratio of the hypo- to hyper-phosphorylated form in pRb-expressing MC3T3 osteoblasts at 14 dbc (Fig. 5C, top right). On the other hand, merlin remained predominantly hyperphosphorylated in pRb-deficient osteoblasts at 14 dbc (Fig. 5C, bottom right). It is noteworthy that appearance of the hypophosphorylated merlin in pRb*-*expressing osteoblasts at 14 dbc coincided with the decreased BrdU incorporation seen in these cells at this time point (Fig. 1E). These observations agree with previous reports showing that hypophosphorylated merlin is the active anti-proliferative form associated with contact-dependent growth arrest [25]. Calf intestinal phosphatase (CIP) treatment of protein lysates confirmed that the merlin doublet is due to phosphorylation, since CIP eliminated the slower-migrating band whereas phosphatase inhibitors reversed this effect (Fig. 5D).

Since merlin attachment to the cell membrane is essential for its function, we tested the effect of pRb on merlin localization. Immunocytochemical localization of merlin revealed two distinct cellular pools in pRb-expressing osteoblasts: a strong punctuated perinuclear staining pattern and a membrane-associated staining (Fig. 5E, left), the latter being consistent with merlin function in adherens junction assembly. In contrast, only the perinuclear staining was apparent in pRb-deficient osteoblasts (Fig. 5E, right), suggesting that merlin becomes detached from adherens junctions in pRb-deficient osteoblasts. To confirm this, merlin was immunoprecipitated from whole cell lysates using a merlin-specific antibody, followed by immunoblotting the precipitates with antibodies against merlin, β-catenin, N-cadherin, and α-tubulin. Merlin was immunoprecipitated as a doublet in both pRb-expressing and pRb-deficient osteoblasts. However, the hypophosphorylated form predominated in pRb-expressing osteoblasts while the hyperphosphorylated form predominated in pRb-deficient osteoblasts (Fig. 5F, top), suggesting merlin inactivation in pRb-deficient osteoblasts. While in pRb-expressing osteoblasts merlin co-immunoprecipitated with adherens junction components β-catenin and N-cadherin, in pRb-deficient osteoblasts it co-immunoprecipitated with α-tubulin (Fig. 5F, second and third panels from top, bottom panel), which is consistent with merlin having a tubulin binding site that is exposed only in the inactive form due to misfolding induced by phosphorylation in Ser518 [26], [27]. Additional studies conducted in the human pRb-deficient osteosarcoma cell line Saos-2 showed that pRb expression is necessary and sufficient to promote merlin activation (Fig. S3).

**Figure 6:**
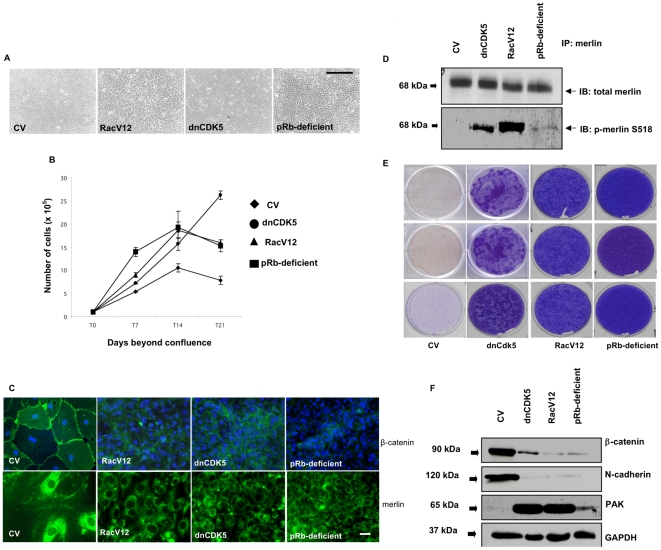
Unrestrained Rac 1 activation in pRb-expressing osteoblasts mimics pRb loss by perturbing cell adhesion. Phase contrast photomicrographs at x10 (**A**) and growth curves (**B**) showing that pRb-expressing MC3T3 osteoblasts stably expressing RacV12 or dnCdk5 grow in culture to a higher density relative to pRb-expressing MC3T3 osteoblasts transfected with control vector (CV). Bar in (**A**)  =  20 µm. Each point in (**B**) represents the mean of 3 independent experiments ± SEM. (**C**) Immunocytochemical analysis showing loss of membrane-associated β-catenin (top) and merlin (bottom) in pRb-expressing MC3T3 osteoblasts transfected with either RacV12 or dnCdk5, as compared to CV-transfected cells. These cells show a staining pattern comparable to that observed in pRb-deficient MC3T3 osteoblasts (last panel to the right). Magnification = ×100. Bar = 1 µm. (**D**) Merlin was immunoprecipitated from whole protein lysates with a merlin-specific antibody followed by immunoblots with a merlin phospho-specific antibody recognizing Ser518. Merlin phosphorylation was observed in pRb-expressing osteoblasts cells transfected with dnCdk5 or Rac V12, as well as in pRb-deficient osteoblasts. (**E**) Triplicates of crystal violet-stained plates showing foci formation in pRb-expressing osteoblasts transfected with dnCdk5 5 or RacV12 and in untransfected pRb-deficient osteoblasts, as compared with CV-transfected cells. (**F**) Immunoblots showing decreased expression of β-catenin and N-cadherin, and increased Pak1 expression in pRb-expressing osteoblasts transfected with dnCdk5 or RacV12, as well as in untransfected pRb-deficient osteoblasts, relative to CV-transfected cells. Experiments were conducted in osteoblasts cultured until 14 dbc.

In order to correlate Rac1 activity with merlin function and adherens junction integrity, we manipulated Rac1 activity in pRb-expressing osteoblasts by transfecting them with plasmid vectors encoding a constitutively activated Rac1 mutant not susceptible to inhibition by pRb (RacV12). We used stable transfectants to investigate the effects of unrestrained Rac1 activity on contact-dependent growth arrest, adherens junction formation, and merlin intracellular localization and phosphorylation. Phase-contrast microscopy (Fig. 6A) and growth curves (Fig. 6B) showed that osteoblasts stably expressing RacV12 grew in culture to a cell density significantly higher than osteoblasts transfected with control vector and comparable to pRb-deficient osteoblasts. A comparable increase in cell density was observed in pRb-expressing osteoblasts transfected with a dominant negative mutant version of the Rac1 repressor the cyclin-dependent kinase 5 (Cdk5) [19], [20], [28], [29].

Using immunocytochemistry, we observed that the membrane staining for β-catenin and merlin is lost in osteoblasts expressing either RacV12 or dnCdk5. Instead, these cells showed a weak, diffuse, and cytoplasmic labeling for β-catenin, whereas merlin staining revealed only the perinuclear form (Fig. 6C). These results show that unrepressed Rac1 activity in pRb-expressing osteoblasts mimics pRb loss by inhibiting merlin function and disrupting adherens junctions.

To assess the phosphorylation status of merlin in pRb-expressing osteoblasts with unrestrained Rac1 activity, merlin was immunoprecipitated from whole protein lysates followed by immunoblotting with a phospho-specific antibody that recognizes merlin when it is phosphorylated in Ser518. Consistent with our hypothesis that pRb promotes merlin activity by blocking Rac1/Pak1, we observed increased merlin phosphorylation in pRb-deficient osteoblasts compared with pRb-expressing osteoblasts transfected with control vector (Fig. 6D). As expected, merlin is highly phosphorylated in pRb-expressing osteoblasts transfected with RacV12 and, consistent with Cdk5s repressive effect on Rac1, in pRb-expressing osteoblasts transfected with dnCdk5 (Fig. 6D). These results show that unrestrained Rac1 activity leads to merlin phosphorylation at Ser518 concomitant with its detachment from the cell membrane. Finally, we observed that cells expressing either dnCdk5 or RacV12 formed crystal violet-stained foci, showing that these cells do not undergo contact-dependent growth arrest (Fig. 6E).

Next, we examined the expression levels of adherens junction components in pRb-expressing osteoblasts stably expressing either dnCdk5 or RacV12. Immunoblots showed that unrestrained Rac1 activity due to the action of dnCdk5 or RacV12 reduced β-catenin and N-cadherin levels (Fig. 6F, top and second from top panels). Consistent with our previous observation that Pak1 is up-regulated in pRb-deficient osteoblasts (see Fig. 5A) and with the hypothesis that pRb blocks Rac1/Pak1 activity, we observed increased Pak1 levels in pRb-deficient osteoblasts compared with pRb-expressing controls (Fig. 6F, third panel from top). Furthermore, Pak1 levels were dramatically increased in pRb-expressing osteoblasts transfected with RacV12 or dnCdk5 (Fig. 6F, *third from top*). These changes are consistent with merlin phosphorylation on Ser518 in pRb-expressing osteoblasts transfected with either dnCdk5 or RacV12 and in pRb-deficient osteoblasts (Fig. 6D). Finally, we observed that blocking Rac1 activity with a dominant negative Rac1 (RacN17) opposed the effects of unrestrained Rac1 activity by partially reestablishing contact-dependent growth arrest and adherens junction formation (Fig. S4).

### Microarray studies show that pRb regulates expression of cell adhesion genes

To test whether pRb regulates cell adhesion-associated gene expression globally, we compared pRb-expressing and pRb-deficient osteoblasts in terms of gene expression patterns using microarray analysis. Microarray data were deposited with the National Center for Biotechnology Information Gene Expression Omnibus (http://www.ncbi.nlm.nih.gov/geo/, accession number GSE19299), and is represented in Tables S1, S2, S3, S4, S5, S6. We used two methods to analyze the microarray data. First, using a canonical pathway database, we performed a cellular pathway analysis that showed that 8 of the top 10 cellular processes affected by pRb are related to cell adhesion, indicating a strong influence by pRb on cell adhesion (Table S1). Second, using the Gene Ontology database to identify cell adhesion-related genes, we obtained 1719 individual probe sets corresponding to genes involved directly or indirectly in cell adhesion. Of these, 1054 (61%) were significantly altered by pRb with a false discovery rate (FDR) of <0.0320 (FDR <0.05 is statistically significant). Table S2 shows cell adhesion genes activated by pRb while table S3 shows cell adhesion genes repressed by pRb. Other approaches such as GeneGo (Table S4), Gene Set (Table S5) and Gene Set Enrichment analyses (Table S6) also ranked cell adhesion high on the list of pRb-regulated processes. Together, these results show that pRb dramatically affects the transcription of a wide array of cell adhesion genes.

### qRT-PCR validated pRb regulation of several genes involved in cell adhesion

**Figure 7:**
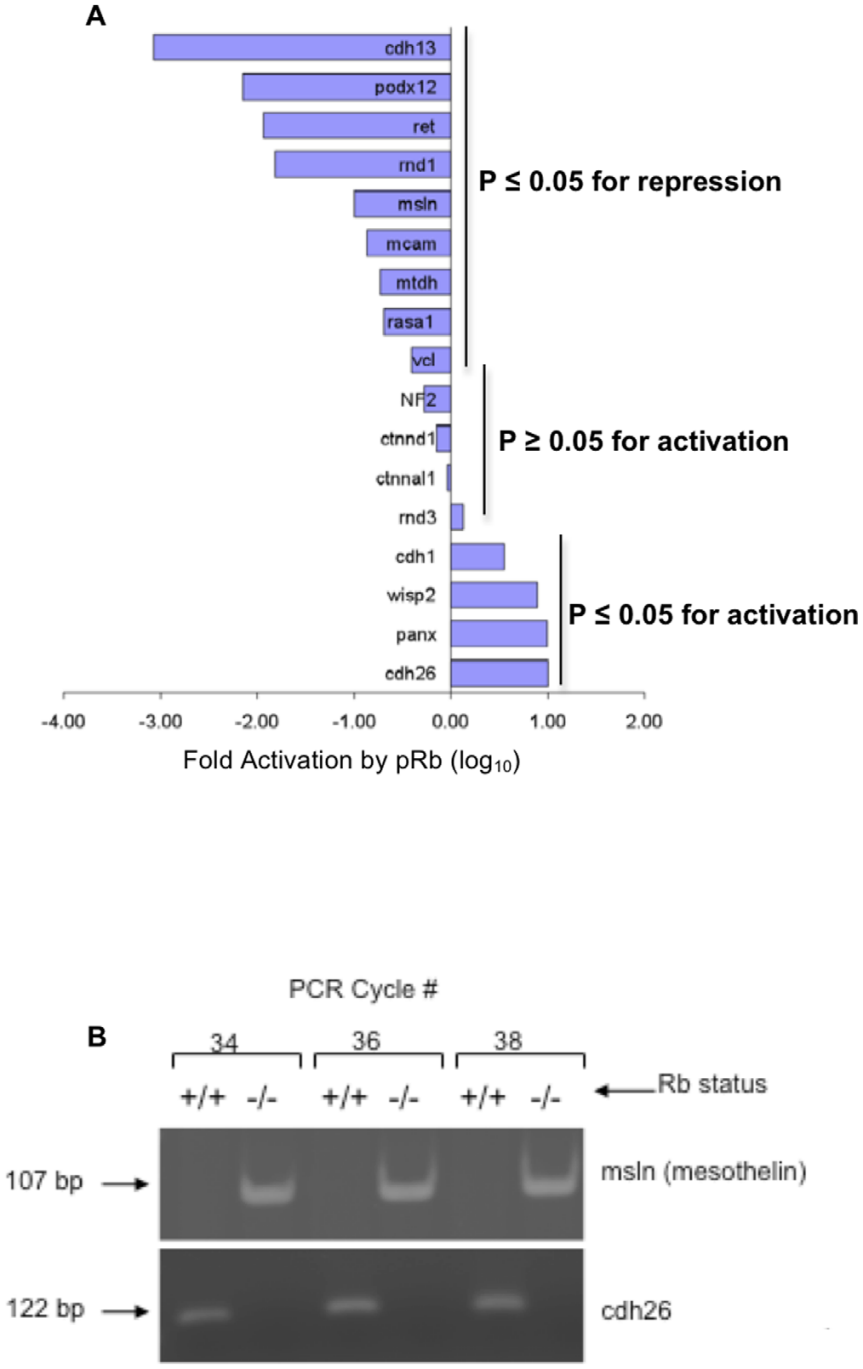
qRT-PCR verified genes identified as pRb-regulated by microarray analysis. (**A**) Eight up-regulated and 9 down-regulated genes were chosen for validation; *x*-axis represents the fold activation of gene expression in the presence of pRb (pRb+/+ osteoblasts divided by pRb−/− osteoblasts). Negative values mean the gene was repressed by pRb, while positive values indicate activation. Each bar corresponds to a different gene. All 9 genes repressed by pRb in microarray analyses were validated (*P*<0.05) while only 4 of the putative pRb-activated genes were validated (*P*<0.05). (**B**) We were unable to perform qRT-PCR on cdh26. Instead, this figure shows a semi-quantitative RT-PCR analysis of mesothelin and cdh26 levels. Results verify that cdh26 is up-regulated and mesothelin is down-regulated by pRb.

Eight up-regulated and 9 down-regulated cell adhesion genes were chosen from the microarray list of pRb-affected cell adhesion genes and validated by qRT-PCR (Fig. 7). Up-regulated genes chosen for validation included those coding for adherens junction components (α- and δ*-catenins, cadherin1/E-cadherin/CDH1, nf2/merlin, cadherin-26,* and *OB-cadherin*). Also chosen for validation was *pannexin 3*, a gap junction protein with tumor suppressive capacity [30]. On the other hand, pRb-repressed genes chosen for validation included cancer cell surface markers involved in promoting invasiveness and metastasis such as melanoma cell adhesion protein (*mcam)*, *mesothelin*, *metadherin*, and *podocalyxin-like 2 (podx12)*, among others [31]–[38]. All 9 of the pRb-repressed genes were validated (*P*<0.05). This was expected given that pRb is a well-characterized transcriptional repressor [39] and gives us confidence in the genes repressed by pRb. Notable among transcripts repressed by pRb according to microarray data was Pak1, validated at both the mRNA and protein levels (Figs. 5A and 5B). In contrast, only 4 of the 8 genes predicted to be up-regulated by pRb (*cadherin1/E-cadherin/CDH1, wisp2, pannexin 3, and cadherin 26*) were validated (P<0.05). *OB-cadherin* (*cadherin-11*) was validated both at the mRNA and protein levels (Figs. 3A and 3B). The other four genes (*nf2/nf2, ctnnd1, ctnnal1* and *rnd3*) were not significantly activated by pRb. This suggests that pRb could be regulating the expression of these genes acting through more complex or indirect mechanisms, for example by controlling the expression, function or DNA binding capacity of transcriptional regulators for these genes.

## Discussion

Carcinogenesis-associated mutations disrupt many aspects of cellular function, cell cycle control and cell adhesion being among them. This raises the possibility that the loss of intercellular adhesion leading to metastasis could also result from the functional disruption of cell cycle regulators at earlier stages of carcinogenesis. Supporting this idea, several reports correlate pRb mutational inactivation with lack of adherens junctions [8]–[10]. Our studies confirm and extend these reports by showing that abrogating pRb expression in murine osteoblasts leads to aberrant expression of surface cadherins, disruption of adherens junctions, and loss of contact-dependent growth arrest. We propose that pRb loss could contribute to tumor formation not only by leading to a breakdown in cell cycle control but also by allowing cells to circumvent the restriction imposed on their proliferation by the establishment of cell-to-cell contacts.

Osteoblastic differentiation strongly depends on cell-to-cell interactions. Osteoblasts originate from pluripotent mesenchymal stem cells that differentiate into stroma, adipocytes, myoblasts, chondroblasts, fibroblasts, or osteoblasts [40], [41]. Stem cells that commit to osteoblastic differentiation are sorted from other mesenchymal cells and align with and adhere to each other. Cadherins mediate these cell-to-cell attachments, and osteoprogenitor cells express a changing repertoire of cadherins that provide cues for their differentiation into mature osteoblasts [12], [42]. Here we showed that adherens junction loss in pRb-null osteoblasts is accompanied by abnormal expression patterns of OB- and N-cadherins, which are the predominant osteoblasts cadherins. This suggests that pRb is required to temporally regulate these changes such that expression of specific cadherins is triggered with the right timing during differentiation. pRb loss could hamper the proper homotypical intercellular contacts, resulting in defective osteoblast differentiation and function with consequent disruption of bone integrity or formation of bone tumors. The structural defects observed in the calvaria of pRb knockout mice support this hypothesis and are suggestive of osteoblasts that are incapable of adhering to each other. Given the central role that both pRb and cell-to-cell interactions play in osteoblast differentiation [4], [15], [43], we postulate that pRb's role in osteoblast differentiation could be related to its capacity to promote the proper cell-to-cell contacts among differentiating osteoblasts.

We showed that pRb-deficient cells have significantly reduced expression of OB-cadherin, which appeared among the transcripts that are up-regulated by pRb in our microarray assays, with an average fold-induction by pRb of 4.45 (P = 0.000). We also found cadherin1/E-cadherin/CDH1 to be up-regulated by pRb. There is a precedent for transcriptional regulation of cadherins by pRb. It has been shown that pRb up-regulates transcription of the *cadherin1/E-cadherin/CDH1* gene via an interaction with the AP-2 transcription factor, a well-known pRb-binding partner and transcriptional co-activator [44]. Supporting this, we found *cadherin1/E-cadherin/CDH1* to be dramatically up-regulated by pRb in our microarray assays, with an average fold-induction of 11.83 over pRb-null cells (P = 0.000, validated by qRT-PCR). However, it remains to be clarified whether other genes found in our microarray studies to be up- or down-regulated by pRb are targets of known pRb binding proteins and transcriptional co-regulators such as AP-2 and E2F. On the other hand, the increase in N-cadherin observed in pRb-deficient osteoblasts is more difficult to explain. N-cadherin did not appear among the transcripts regulated by pRb in our microarrays, suggesting that a regulation of N-cadherin by pRb is indirect via other factors. However, this change in cadherin expression is strikingly reminiscent of the cadherin switch occurring during the epithelial-to-mesenchymal transition (EMT) that is part of oncogenic transformation. Indeed, pRb abrogation in several epithelial cell lines has been shown to induce changes associated with an EMT, including such a cadherin switch in which cadherin1/E-cadherin/CDH1 is replaced by N-cadherin [45]-[47]. While a cadherin switch in epithelial cells could trigger metastasis, a similar switch in osteoblasts could profoundly disrupt bone morphogenesis and promote osteosarcoma formation by affecting the cell-to-cell interactions that drive osteogenic differentiation. Our data showing a switch from OB- to N-cadherin upon pRb loss are fully consistent with other reports and lend credence to the hypothesis that pRb could prevent cancer spreading and metastasis by promoting cell-to-cell adhesion.

pRb appears to have a global influence on cell adhesion beyond the control of cadherin genes. Our microarrays show that pRb controls expression of a wide range of cell adhesion genes, thus having a strong influence over cellular processes related to adhesion. We observed that pRb promotes the expression of genes involved in the promotion of adhesion of differentiated cells while repressing the expression of genes associated with invasiveness and metastasis and whose products could be used by cancer cells to engage in transient interactions along their migration pathways. Furthermore, our microarray data show that pRb up-regulates the expression of genes coding for proteins involved in several types of interactions between the cell and its surroundings, such as integrins and gap and tight junction proteins.

**Figure 8:**
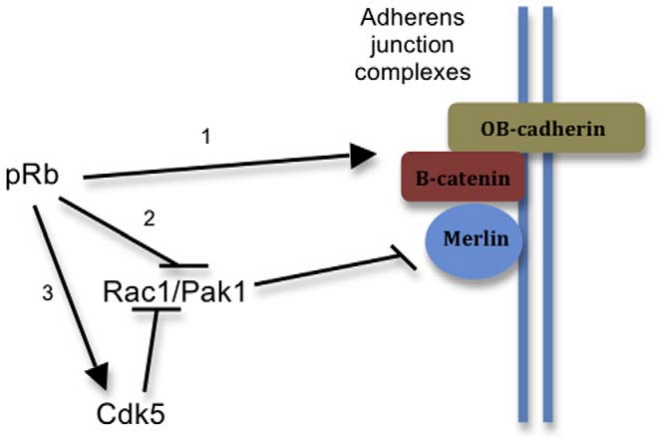
Possible mechanisms by which pRb could regulate cell adhesion. pRb regulates transcription of adherens junction and cell adhesion genes (*1*). pRb also regulates cell adhesion by repressing Rac1, a repression that allows merlin activation and adherens junction assembly. pRb achieves this by down-regulating the expression of Pak1 (*2*) or by activating Cdk5 (*3*). The relevance of (*3*) for the pRb-mediated assembly of adherens junctions is still unclear.

Our data also suggest a model integrating pRb, Rac1, Pak1, and merlin in adherens junction assembly (Fig. 8). pRb could promote adherens junction assembly by regulating the expression of adherens junction genes and/or by repressing Rac1 activity. pRb can repress Rac1 by down-regulating Pak1 levels or, alternatively, via Cdk5, a known Rac1 repressor [19], [20], [28], [29]. Since Cdk5 activity can be triggered by pRb in osteoblasts [19], [20], we used dnCdk5 as an additional tool to induce unrestrained Rac1 activity, and it yielded results that are indistinguishable from pRb deletion or transfection with RacV12. However, we have no conclusive data showing that Cdk5 intervention is necessary for the pRb-dependent repression of Rac1/Pak1.

Although we have shown that abrogating pRb function in osteoblasts has a disruptive effect on cell adhesion, it remains to be established whether altering the function of upstream components of the pRb pathway such as CDK4, cyclin D, and p16ink4a, will have similar consequences for intercellular adhesion. At this moment it is unclear whether a function on cell adhesion is unique for pRb or if it is shared with other components of the pRb pathway or even with the other pocket proteins p107 and p130. This is an important question that must be addressed in follow-up studies since mutations in CDK4, cyclin D, and p16ink4a are linked to the molecular etiology of a wider spectrum of human tumors than mutational inactivation of pRb itself [48]. Given the intimate functional interrelatedness among these proteins, it would be expected that loss of p16ink4a with concomitant pRb inactivation, for example, would be functionally equivalent to pRb loss. Interestingly, studies on the molecular etiology of lung cancer have shown that the consequences of mutating pRb could differ profoundly, both biochemically and in terms of phenotype, from the consequences of mutating p16ink4a. For example, although it is accepted that inactivation of only one component of the pRb pathway is required for lung tumorigenesis, the subset of tumors that arises as a consequence of pRb loss is different from the one that arises due to p16ink4a loss [48]. This illustrates that inactivating different components of the same pathway does not necessarily results in the same phenotype.

In summary, this study contributes to the characterization of a non-traditional role for pRb as a regulator of cell adhesion and provides mechanistic insights for this role. Conceptually, a dual role for pRb as a regulator of both cell cycle progression and adherens junction assembly could be of central importance in the orchestration between cell proliferation and cell adhesion, an orchestration that may be pivotal for tissue morphogenesis. A disruption of pRb function with the resulting breakdown of this orchestration could be at the core of the molecular etiology of some human cancers. This dual role for pRb implies that pRb inactivation could play a significant role not only during initial tumor formation by extending the replicative capacity of mutant cells but also at later stages of tumorigenesis, by disrupting cell-to-cell interactions consequently leading to metastasis. This dual role for pRb could contribute to what has been described by some cancer researchers as “the hope to achieve an understanding of the complex process of neoplastic transformation at the cellular level in terms of a small number of genetic changes” [49].

## Materials and Methods

### Ethics statement

All animal studies were conducted using procedures approved by the Institutional Animal Care and Use Committee of Tufts University & Tufts Medical Center and covered under protocol number B2009-124.

### Animals

The flox19-RB1 mice were obtained from the laboratory of Doug Hanahan (San Francisco, CA) and maintained in a C57BL/6 background. 2.3- and 3.6-Col1a1-Cre transgenic mice were obtained from the laboratory of Barbara Kream (Farmington, CT). The F1 RBf19/WT;Col1a1-Cre mice were self-crossed and the resulting F2 mice were used in our studies.

### Calvarial cell preparation and cultures

Primary osteoblasts were isolated from embryonic day 18.5 (E18.5) mice calvarial bone. Briefly, calvariae (topmost skull bones) were removed, rinsed with PBS, and digested in 0.05% Trypsin/EDTA plus 0.1% collagenase P (Boehringer Mannheim) at 37°C with shaking. Cell suspensions were collected from the digestion reaction at 10-min intervals and centrifuged at 1,600 g for 5 minutes. The resulting cell pellet was resuspended in minimum essential medium-alpha (α-MEM) (GIBCO®/Invitrogen) supplemented with 10% fetal bovine serum (FBS, Invitrogen) and 1% penicillin-streptomycin (Sigma), and plated in 6-cm culture plates. Osteoblast differentiation was induced by culturing them in the presence of 0.01 M β-glycerophosphate, 100 mM I-ascorbic acid and 0.1 µM dexamethasone (Sigma). 3T3-immortalized osteoblasts (MC3T3 cells) were obtained by culturing primary osteoblasts using the 3T3 protocol, in which 3×10^5^ cells were plated in 6-cm culture plates and passaged every 3 days. The population doubling level during each passage was calculated according to the formula log (final cell number/3×10^5^)/log2. MC3T3 cells were maintained in α-MEM supplemented with 10% FBS and 1% penicillin-streptomycin.

### Plasmids and transfections

Plasmids pEBG-RacV12 and pEBG-RacN17 (from the laboratory of John Blenis) have been previously described [50]. Plasmid pCDNA3-dnCdk5 was a gift from Li-Huei Tsai. MC3T3 cells were transfected at 70-80% confluence with FuGENE® (Roche) following manufacturer's specifications, selected with 0.5 µg/ml puromycin, and maintained under selection medium for the duration of the experiments.

### Growth curves

Cells were plated in triplicate at an initial density of 10^5^ cells per 6-cm culture plate. Cells were trypsinized and counted on a weekly basis for 3 consecutive weeks.

### Focus and colony assays

To determine focus formation, MC3T3 cells were plated at a density of 10^5^ cells per 6-cm culture plate, grown to confluence, and maintained without splitting for 14–21 days. Medium was changed every 3 days. Cultures were then rinsed with PBS and stained with 4 mg/ml crystal violet in 10% methanol. For colony formation in soft agar, MC3T3 cells were plated at the same density as described above in 0.4% Sea Plaque® low-gelling temperature agarose (American Bioanalytical) in α-MEM supplemented with 10% FBS. The number of colonies per visual field was scored 21 days later.

### Tumorigenicity assay

For *in vivo* tumor formation, 1×10^6^ MC3T3 cells were injected into flanks of 4- to 6-week-old SCID/NCr (BALB/C) female mice (National Cancer Institute). Three mice were used for each cell type tested. Tumor formation was scored 3 weeks after injection.

### BrdU immunocytochemistry

Cells grown in chambered tissue culture slides were cultured for 24 h in the presence of 10 µM BrdU before being processed for BrdU immunocytochemistry at various time points. We used the BrdU in situ detection kit (BD Pharmingen), according to manufacturers specifications. Briefly, cells were fixed for 15 minutes in 2% paraformaldehyde/0.2% glutaraldehyde in PBS and permeabilized for 30 minutes in 1% bovine serum albumen (BSA)/0.2% Triton X-100 in PBS, followed by incubation in BD Retrieve Antigen solution A at 89°C for 10 minutes. Chamber slides were cooled to room temperature, washed 3 times with PBS, and incubated for 1 h at room temperature with biotinylated anti-BrdU antibody diluted 1∶10 in 1% BSA/0.2% Triton X-100 in PBS. Slides were then washed 3 times in PBS and incubated for 30 minutes at room temperature with streptavidin-conjugated Alexa 499 diluted 1∶20 in 1% BSA/0.2% Triton X-100 in PBS. Slides were washed again 3 times with PBS, counterstained with Hoechst 33258 for 1 minute, rinsed 5 times with distilled water, and mounted in Fluoromount-G (Southern Biotech).

### Alizarin red-S staining of coronal skull sections and osteoblast cultures

Paraffin-embedded skull coronal sections obtained from E18.5 mice were deparaffinized by heating at 58°C–60°C for 30 minutes, followed by three 2-min washes in xylene. Sections were rehydrated with a series of washes of 100%, 95%, 70%, and 50% ethanol, followed by a final wash in distilled water. They were then stained in a 0.5% alizarin red-S solution (pH 4.2) for 5–30 minutes, rinsed 3–5 times with distilled water, allowed to air-dry, and mounted with Cytoseal 60 (Stephens Scientific). Staining of cultures was done by incubating in 0.5% alizarin red-S solution for 5 minutes, followed by rinsing with distilled water and air-drying.

### Western immunoblotting

Protein expression was detected by immunoblotting following standard procedures. Cells were lysed in 100–200 µL of ELB (50 mM HEPES, pH 7.2, 250 mM NaCl, 2 mM EDTA, 0.1% NP-40, 1 mM dithiothreitol) plus protease and phosphatase inhibitors (1 mg/ml of aprotinin, 1 µg/ml of leupeptin, 100 µg of phenylmethylsulfonyl fluoride, 4 mM sodium orthovanadate, 2 mM sodium pyrophosphate). Lysate protein concentration was determined by BioRad protein assay. For immunoblotting, 50-100 µg of protein were separated by SDS-PAGE, transferred to nitrocellulose, and immunoblotted with the indicated primary antibody. Secondary antibodies used were horseradish peroxidase-conjugated donkey anti-mouse or donkey anti-rabbit (Jackson Immunosciences). For immunoprecipitations, protein lysates (100–200 µg) were immunoprecipitated with the indicated antibody overnight at 4°C, protein A-sepharose CL-4B beads (30 µL Amersham Biosciences) were added for 1 h, and following centrifugation beads were then washed four times with ELB lysis buffer and separated by SDS-PAGE. For calf intestinal phosphatase (CIP) treatment, protein lysates (50 µg) were treated with 50 units of CIP overnight at 37°C and then separated by SDS-PAGE. The CIP inhibitors used were 20 mM Na_3_VO_4_, 2 mM sodium pyrophosphate, and 40 mM sodium fluoride.

### Immunofluorescence

Cells grown in chambered culture slides were rinsed 3 times with cold PBS, fixed in methanol for 10 minutes on ice, and allowed to air-dry. After another rinse in PBS, cells were blocked with 10% BSA in PBS for 30 minutes at 4°C, rinsed again in PBS, incubated for 1 h at room temperature with primary antibody diluted 1∶100 in PBS containing 1% BSA and 0.2% Triton X-100, and then briefly rinsed with cold PBS and incubated for 1 h at room temperature with either Alexa Fluor 546-conjugated goat anti-rabbit or Alexa Fluor 488-conjugated goat anti-mouse secondary antibody (Molecular Probes) diluted 1∶200 in PBS. DNA was stained with Hoechst 33258 for 1 minute. Cells were then rinsed 5 times with distilled water and mounted with Fluoromount-G. We used a Nikon Eclipse 80i microscope to observe slides and a SPOT RT KE camera using the SPOT software version 4.1 for Mac for image acquisition.

### Small interfering RNA

A short hairpin-interfering RNA targeting the *RB* gene (*RB*-shRNA) was designed and cloned using the siSTRIKE U6 Hairpin Cloning System from Promega, following manufacturer's instructions. We used the siRNA Target Designer (www.promega.com/siRNADesigner/) to design oligonucleotides using the *RB* gene as the input sequence. The following oligonucleotides targeting position 3168–3186 within the *RB* gene were synthesized (Integrated DNA Technologies): 5′-accggaccctaacacagtatatttcaagagaatatactgtgttagggtcctttttc-3′ and 5′-tgcagaaaaaggaccctaacacagtatattctcttgaaatatactgtgttagggtc-3′. Oligonucleotides were annealed, ligated and cloned in the psiSTRIKE™ vector. Constructs were transfected as described above.

### Immunohistochemistry

E18.5 skull coronal sections were deparaffinized and rehydrated as described above. After rehydration, tissue sections were incubated for 30 minutes in 0.3% H_2_O_2_ in PBS to inactivate endogenous peroxidase activity, incubated for 15 minute at 37°C in 4 N HCl for antigen retrieval, blocked for 1 h at room temperature in 3% BSA in PBS, and then incubated with primary antibody in a humid chamber for 3 h at room temperature. Primary antibodies were diluted 1∶100 in 3% BSA in PBS. Tissue sections were then washed 3 times in PBS and incubated for 2 h at room temperature with horseradish peroxidase-conjugated donkey anti-mouse or donkey anti-rabbit secondary antibodies (Jackson Immunosciences), followed by 4–5 washes in PBS and then incubated with 3,3′-diaminobenzidine in 0.05 M Tris-HCl (pH 7.6) for 5–30 minutes until the appearance of a dark brown precipitate, followed by hematoxylin counterstaining for 1 minute and 3–4 washes in distilled water. Tissue sections were then allowed to air dry for 5 minutes and mounted with Cytoseal 60. Antibodies used for immunoblotting, immunofluorescence, and immunohistochemistry were anti-β-catenin (H-102 Santa Cruz, sc-7199), anti-N-cadherin (BD Transduction Laboratories, #610920), anti-pRb (BD Pharmingen, #554136), anti-PAK (N-20 Santa Cruz, sc-882), anti-Rac1 (C-11 Santa Cruz, sc-95), anti-OB-cadherin (H-50 Santa Cruz, sc-28643), anti-ezrin (H-276 Santa Cruz, sc-20773), anti-merlin (C-18 Santa Cruz, sc-332; and A-19, Santa Cruz, sc-331), anti-α-tubulin (Oncogene, #CP06), and anti-GAPDH (Calbiochem #CB1001). The merlin phospho-specific antibody was provided by Joseph Kissil.

### Quantitative real-time RT-PCR

Total RNA was extracted and purified using the RNeasy kit (Qiagen, Valencia, CA), followed by DNase treatment. One µg of RNA was reverse transcribed using the iScript cDNA synthesis kit (BioRad, Hercules, CA), according to manufacturer's instructions. Pak1, Rac1, OB-cadherin, N-cadherin, and β-catenin mRNA expression levels were determined using TaqMan® Assay (Applied Biosystems, Carlsbad, CA). Briefly, 50 ng of cDNA were subjected to 50 cycles of quantitative PCR in an iCycler (BioRad) using TaqMan® Universal Mastermix according to the manufacturers' instructions. Samples were normalized to the GAPDH reference gene and relative expression levels of all genes were determined using the ^ΔΔ^Ct method. TaqMan® assays used for these studies were: Pak1 (Mm0044612_m1), Rac1 (Mm00488847_m1), OB-cadherin (Mm00515462_m1), N-cadherin (Mm00483213_m1), β-catenin (Mm00499427_s1), and GAPDH (Mm00483213_m1).

### Sample processing for microarray analysis

A mouse genome 430 2.0 microarray chip was used for this study. Five micrograms of total RNA from each sample were processed for microarray analysis. Samples were generated and processed in triplicate. Poly(A) RNA was converted to cDNA and then amplified and labeled with biotin as described by Van Gelder et al. [51]. Hybridization with the biotin-labeled RNA, staining, and scanning of the chips followed the prescribed procedure outlined in the Affymetrix technical manual. Microarray data are MIAME compliant and the raw data were deposited in the National Center for Biotechnology Information Gene Expression Omnibus (GEO) (http://www.ncbi.nlm.nih.gov/geo/), which is a MIAME compliant database as detailed in the MGED Society website (http://www.mged.org/Workgroups/MIAME/miame.html). Accession number for our microarray data is GSE19299.

### Microarray data analysis

Scanned output files were visually inspected for hybridization artifacts and then analyzed using Affymetrix Microarray 5.0 software. Signal intensity was scaled to a trimmed mean intensity of 500 (MAS5) prior to output. Microarray data quality, checked by Chip-wise Correlation Plot [52], was found to be satisfactory. Statistical analyses for individual genes and pathways were performed on 1719 cell adhesion-related genes (Gene Ontology database, http://www.geneontology.org) as well as the Chip-wise genes. MetaCore (version 5.3 build 18499, GeneGo, Hollywood, FL), SAM and SAM-GSA software (http://www-stat.stanford.edu/~tibs/SAM/), and Gene Set Enrichment Analysis (GSEA, http://www.bioconductor.org/) were used to analyze individual genes and gene sets. For individual genes, tests of statistical significance between wild-type and pRb-deficient samples were conducted using Wilcoxon tests; permutation number was set to no less than 500, false discovery rate (FDR) was strictly set under 5%, and fold change was used to determine the quantity of genes included in the final outcome. Annotations of multiple gene databases were downloaded from the Affymetrix website according to the chip model (see supplemental materials). Genes of interest were selected from output genes. We determined qPCR assay eligibility by using UCSC genome browser to confirm specific position, transcriptional direction and consensus of multiple databases, including RefSeq, Uniprot, GenBank, and Comparative Genomics. Target gene sequences for qPCR were obtained from NetAffx module of Affymetrix website. For cellular pathway analysis, MetaCore is based on hypergeometric distribution, SAM-GSA uses “Maxmean” statistic, and GSEA uses modified Kolmogorov-Smirnov statistic. Gene sets were retrieved from http://www.broad.mit.edu/gsea/msigdb/collections.jsp, and biological process ontology subset was selected based on information described in http://www.geneontology.org/GO.process.guidelines.shtml.

### Validation of microarray data

Microarray data were validated with quantitative real-time quantitative PCR (qRT-PCR), performed using the BioRad iQ SYBR Green Supermix on a MyiQ Single Color real-time PCR detection system. Primers used are available upon request. Selected genes from the microarray analysis were validated by qRT-PCR. Briefly, RNA prepared for microarray from MC3T3 Rb+/+ and MC3T3 Rb−/− cells was reverse transcribed using iScript cDNA synthesis kit from BioRad. The cDNA was diluted 1∶30 and qRT-PCR was performed with 1.5–3.0 µL cDNA in a 25-µL final volume in triplicates on 96-well plates, using BioRad SYBR green on the MyiQ PCR detection system. We used the following mouse primer pairs: cdh13, 5′-cagaatcaatgagaacacagg-3′ (f) and 5′-atcacaatgacttccagagg-3′ (r); podxl2, 5′-tgggaagaagaggaactaaa-3′ (f) and 5′-ctcagtcaggtctgggaag-3′ (r); ret, 5′-tggcacacctctgctctatg-3′ (f) and 5′-gcggatccagtcattctcat-3′ (r); rnd1, 5′-tgtcccaccagaagcaggcaccca-3′ (f) and 5′-cggaagtgaaagccgagccctccaggt-3′ (r); msln, 5′-aggatagccttgtgggtag-3′ (f) and 5′-aacgagattcccttcaccta-3′ (r); cdh26, 5′-tgggatggcaacgaggaggg-3′ (f) and 5′-acaacgacgaggctatgggc-3′ (r); panx3, 5′-cccccaagtccccattctcagcagc-3′ (f) and 5′-tgcggtcaggcagcagggca-3′ (r); wisp2, 5′-gtggtgctgtgtgcctctt-3′ (f) and 5′-aaggtctccccatccaggta-3′ (r); *cadherin1/E-cadherin/CDH1*, 5′-gctggaccgagagagttacc-3′ (f) and 5′-tgttgtgctcaagccttcac-3′ (r); rnd3, 5′-cgtccgtccactctcttaccca-3′ (f) and 5′-tgcagcccaccaacagcatc-3′ (r); ctnnal1, 5′-tggcgagggctgtgcttgaa-3′ (f) and 5′-tccgtgaccttctccagggc-3′ (r); ctnnd1, 5′-agactgggctattctctgtg-3′ (f) and 5′-gggcaggataaaaagtaagg-3′ (r); nf2, 5′-ggggctaagagacccagaac-3′ (f) and 5′-aagaaccaggtttcccgaag-3′ (r); vcl, 5′-gcacatctgacctactgctt-3′ (f) and 5′-ccactacctctgccactgta-3′ (r); rasa1, 5′-gcgtttttcttcactctcag-3′ (f) and 5′-cgccttctatcttctactgg-3′ (r); mtdh, 5′-gttaccaccgagcaacttac-3′ (f) and 5′-agattttcattcagccttga-3′ (r); mcam, 5′-aaactggtgtgcgtcttct-3′ (f) and 5′-tactggctgcttttcctct-3′ (r).

## Supporting Information

Figure S1 pRb-deficient osteoblasts show traits of the transformed phenotype. (a) To determine if pRb-deficient MC3T3 osteoblasts are able to form tumors in vivo, 1×106 cells were injected subcutaneously into SCID/NCr BALB/C mice. Three weeks after injection, mice injected with pRb-deficient MC3T3 osteoblasts developed highly vascularized tumors (arrow), while no tumors were apparent in mice injected with pRb-expressing MC3T3 controls. (b) While pRb-expressing MC3T3 osteoblasts were unable to grow in soft agarose and remained as single cells (left), pRb-deficient MC3T3 osteoblasts were able to proliferate to form cell colonies in soft agar (right), indicating that they have the capacity to grow in an anchorage-independent manner. Magnification is 10X, bar = 20 µm.
(6.32 MB TIF)

Figure S2 Knock-down of endogenous pRb in pRb-expressing osteoblasts disrupts adherens junctions. A DNA construct encoding a short hairpin-interfering RNA targeting the mouse RB gene (RB-shRNA) was cloned into an expression vector. pRb-expressing MC3T3 cells were then transfected with this construct followed by selection of stable transfectants. (a). Phase contrast micrographs at 4x showing that pRb-expressing MC3T3 in which pRb expression is suppressed by RB-shRNA (right) grow to a higher cell density when compared to pRb-expressing MC3T3 cells transfected with control vector (left). Bar = 2 µm. (b) Immunocytochemical localization of pRb and β-catenin in pRb-expressing MC3T3 osteoblasts transfected with control vector (left) showed nuclear immunoreactivity for pRb (green) and membrane-associated immunoreactivity for β-catenin (red). Immunocytochemical analysis of pRb-expressing MC3T3 cells transfected with the RB-shRNA (middle) showed a strong correlation between pRb and β-catenin expression in these cultures, pRb and β-catenin expression being undetectable in the same cells. Total nuclei stained with DAPI (blue) are shown in the right panel, magnification is 100×, bar = 1 µm.
(6.53 MB TIF)

Figure S3 pRb regulates merlin folding in human osteoblasts. To determine if pRb expression is necessary and sufficient to promote merlin activation, we investigated merlin-tubulin interactions in the pRb-deficient osteosarcoma cell line Saos-2. Saos-2 cells were transfected with either control vector (CV) or with a vector transducing pRb. Cells transfected with control vector and pRb-transfected cells were cultured for 2, 5, 7, and 10 days following transfection and harvested to obtain whole protein lysates. Merlin was immunoprecipitated from these lysates using a merlin-specific antibody, followed by immunoblot analysis using antibodies against merlin and α-tubulin. Merlin is predominantly co-immunoprecipitated with α-tubulin in CV-transfected cells, while this interaction is significantly diminished as early as 2 days after pRb reintroduction, suggesting that merlin is activated soon after pRb reintroduction into Saos-2 cells.
(6.53 MB TIF)

Figure S4 Blocking Rac1 activity reestablishes adherens junction formation and contact-dependent growth arrest. In these experiments, pRb-expressing osteoblasts stably expressing dnCdk5 were transfected with either a control vector or a dominant negative Rac1 (RacN17), followed by selection of stable transfectants. A–B. Phase contrast photomicrographs at 4× magnification (bar = 2 µm) (A) and growth curve analysis (B) showing that pRb-expressing osteoblasts stably expressing dnCdk5 and RacN17 grew in culture to a lower cell density than dnCdk5-expressing osteoblasts transfected with control vector (CV). In each graph each data point represents the mean of three independent experiments ± standard error. (c) Immunocytochemical localization of β-catenin showing that its membrane-associated localization is partially reestablished in pRb-expressing osteoblasts expressing both dnCdk5 and RacN17. Magnification is 100×, bar = 1 µm. (d) Crystal violet staining showing reduced number of foci in pRb-expressing osteoblasts expressing both dnCdk 5 and RacN17.
(2.30 MB TIF)

Table S1 pRb deficiency dramatically affects the expression of genes involved in cell adhesion pathways in osteoblasts. MetaCore cellular pathway analysis in conjunction with the Canonical Pathway database from GeneGO Inc. were used to determine which canonical pathways are statistically affected by pRb deficiency. This analysis allowed us to determine the relative position of cell-adhesion-related cellular pathways within all the cellular pathways regulated by pRb. Analyses based on the GeneGO database yielded 139 cellular processes (out of 289 total cellular processes) that were significantly affected by pRb expression (P>0.05 and false discovery rate (FDR) <0.0175). Remarkably, 8 of the top 10 cellular processes affected by pRb expression are all related to cell adhesion. Blue shading indicates the cut off of statistical significance. This table summarizes results from several microarray analyses, all of which indicate that pRb dramatically affects expression of genes involved in cell adhesion. It is noteworthy that cell cycle does not appear prominently in the pathways regulated by pRb in these cells. This may not come as a surprise as the cell lines used (pRb-expressing and pRb-deficient) were harvested during the proliferative phase of growth, thus minimizing the well-known effects of pRb on cell cycle progression.
(0.26 MB XLS)

Table S2 Cell Adhesion Related Genes Positively Regulated by Rb. Gene Ontology database prioritization for cell adhesion genes yielded 1719 individual probe-sets corresponding to genes designated to be involved directly or indirectly in cell adhesion. This table shows those that were positively regulated by pRb. For these 1719 individual probesets, tests for magnitudes in changes in gene expression between pRb-expressing and pRb-deficient osteoblasts as well as for statistical significance of these changes were conducted using SAM software running the Wilcoxon algorithm. Out of 1719 cell-adhesion related probe-sets, 1054 (61%) were significantly altered by pRb with a false discovery rate (FDR) of <0.0320 (FDR <0.05 is considered statistically significant). Of these 1054 genes, 462 (27%) were altered at least two-fold by pRb (FDR = 0.0219). As an example, OB-cadherin (encoded by the cdh11 gene) is among these genes and is down-regulated 4.45-fold in pRb-deficient cells. One-hundred and thirty-seven genes (8%) and 63 genes (4%) were altered by at least 5- and 10-fold, with FDRs of 0.0041 and 0.0001, respectively, by pRb expression. Together, these results show that pRb dramatically affects the transcription of a wide array of cell adhesion genes.
(0.48 MB XLS)

Table S3 Cell Adhesion Related Genes Negatively Regulated by Rb. Description for this table is the same as for Table S2, except that this table highlights genes repressed by pRb.
(0.33 MB XLS)

Table S4 GeneGo Analysis. Analyses based on the GO database from the GO project confirmed the results from the Canonical Pathway Database shown in table S1 by ranking a cell-adhesion process with the second best P value among a total of 3752 GO functional processes. These data demonstrate, in an arbitrary fashion, that cell adhesion processes are among those most significantly affected by pRb.
(1.71 MB XLS)

Table S5 Gene Set Analysis (GSA, one module of SAM). GSA was performed using GO Project database. Results indicated that pRb knockout would significantly affect adhesion related processes.
(0.01 MB XLS)

Table S6 Gene Set Enrichment Analysis (GSEA, one module of SAM). GSEA was performed using GO Project database. Results again indicated that pRb knockout would significantly affect adhesion related processes.
(0.01 MB XLS)

## References

[1] Weinberg RA (1995). The Rb protein and cell cycle control.. Cell.

[2] Dyson N (1998). The regulation of E2F by pRB-family proteins.. Genes Dev.

[3] Ross JF, Liu X, Dynlacht BD (1999). Mechanism of transcriptional repression of E2F by the retinoblastoma tumor suppressor protein.. Mol Cell.

[4] Thomas DM, Carty SA, Piscopo DM, Lee J-S, Wang W-F (2001). The retinoblastoma protein acts as a transcriptional coactivator required for osteogenic differentiation.. Mol Cell.

[5] Thomas DM, Yang H-S, Alexander K, Hinds PW (2003). Role of the retinoblastoma protein in differentiation and senescence.. Cancer Biology & Therapy.

[6] Knudsen ES, Knudsen KE (2006). Retinoblastoma tumor suppressor: where cancer meets cell cycle.. Experimental Biology and Medicine.

[7] Du W, Pogoriler J (2006). Retinoblastoma family genes.. Oncogene.

[8] van Aken EH, Papeleu P, De Potter P, Bruyneel E, Philippé J (2002). Structure and function of the N-cadherin/catenin complex in retinoblastoma.. Invest Ophthalmol Vis Sci.

[9] Kashima T, Kawaguchi J, Takeshita S, Kuroda M, Takanashi, M (1999). Anomalous cadherin expression in osteosarcoma: possible relationships to metastasis and morphogenesis.. Am J Pathol.

[10] Rodríguez-Salas N, Palacios J, de Castro J, Moreno G, González-Baron M (2001). Beta-catenin expression pattern in small cell lung cancer: correlation with clinical and evolutive features.. Histol Histopathol.

[11] Haÿ E, Lemonnier J, Modrowski D, Lomri A, Lasmoles F (2000). Cadherins mediate early human osteoblast differentiation promoted by BMP-2.. J Cell Physiol.

[12] Cheng S-L, Shin CS, Towler DA, Civitelli R (2000). A dominant negative cadherin inhibits osteoblast differentiation.. J Bone Mineral Research.

[13] Marie P (2002). Role of N-cadherin in bone formation.. J Cell Physiol.

[14] Stains JP, Civitelli R (2005). Cell interaction in regulating osteogenesis and osteoblast function.. Birth Defects Research.

[15] Gutierrez GM, Kong E, Sabbagh Y, Brown NE, Lee JS (2008). Impaired bone development and increased mesenchymal progenitor cells in calvaria of RB1-/- mice.. Proc Natl Acad Sci U S A.

[16] Khanna C, Wan X, Bose S, Cassaday R, Olomu O (2004). The membrane-cytoskeleton linker ezrin is necessary for osteosarcoma metastasis.. Nat Med.

[17] Curto M, McClatchey AI (2004). Ezrin...a metastatic detERMinant?. Cancer Cell.

[18] Braga VM, Betson M, Li X, Lamarche-Vane N (2000). Activation of the small GTPase Rac 1 is sufficient to disrupt cadherin-dependent cell-cell adhesion in normal human keratinocytes.. Mol Biol Cell.

[19] Yang H-S, Hinds PW (2003). Increased ezrin expression and activation by CDK5 coincident with acquisition of the senescent phenotype.. Mol Cell.

[20] Alexander K, Yang H-S, Hinds PW (2004). Cellular senescence requires CDK5 repression of Rac 1 activity.. Mol Cell Biol.

[21] Lallemand D, Curto M, Saotome I, Giovannini M, McClatchey AI (2003). *NF2* deficiency promotes tumorigenesis and metastasis by destabilizing adherens junctions.. Genes Dev.

[22] Shaw RJ, Páez JG, Curto M, Yaktine A, Pruitt WM (2001). The Nf2 tumor suppressor, merlin, functions in Rac-dependent signaling.. Dev Cell.

[23] Kissil JL, Johnson KC, Eckman MS, Jacks T (2002). Merlin phosphorylation by p21-activated kinase 2 and effects of phosphorylation on merlin localization.. J Biol Chem.

[24] Xiao G-H, Beeser A, Chernoff J, Testa JR (2002). p21-activated kinase links Rac/cdc42 signaling to merlin.. J Biol Chem.

[25] Shaw RJ, McClatchey AI, Jacks T (1998). Regulation of the neurofibromatosis type 2 tumor suppressor protein, merlin, by adhesion and growth arrest stimuli.. J Biol Chem.

[26] Xu H-M, Gutmann DH (1998). Merlin differentially associates with the microtubule and actin cytoskeleton.. J Neurosci Res.

[27] Gutmann DH, Hirbe AC, Haipek CA (2001). Functional analysis of neurofibromatosis 2 (NF2) missense mutations.. Hum Mol Genet.

[28] Nikolic M, Chou MM, Lu W, Mayer BJ, Tsai L-H (1998). The p35/Cdk5 kinase is a neuron-specific Rac effector that inhibits Pak1 activity.. Nature.

[29] Rashid T, Banerjee M, Nikolic M (2001). Phosphorylation of Pak1 by the p35/Cdk5 kinase affects neuronal morphology.. J Biol Chem.

[30] Penuela S, Bhalla R, Gong XQ, Cowan KN, Celetti SJ (2007). Pannexin 1 and pannexin 3 are glycoproteins that exhibit many distinct characteristics from the connexin family of gap junction proteins.. J Cell Sci.

[31] Ito T, Maki N, Hazeki O, Sasaki K, Nekooki M (2007). Extracellular and transmembrane region of a podocalyxin-like protein 1 fragment identified from colon cancer cell lines.. Cell Biol Int.

[32] Brown DM, Ruoslahti E (2004). Metadherin, a cell surface protein in breast tumors that mediates lung metastasis.. Cancer Cell.

[33] Wu GJ, Fu P, Wang SW, Wu MW (2008). Enforced expression of MCAM/MUC18 increases in vitro motility and invasiveness and in vivo metastasis of two mouse melanoma K1735 sublines in a syngeneic mouse model.. Mol Cancer Res.

[34] Gould-Rothberg BE, Bracken MB, Rimm DL (2009). Tissue biomarkers for prognosis in cutaneous melanoma: a systematic review and meta-analysis.. J Natl Cancer Inst.

[35] Zabouo G, Imbert AM, Jacquemier J, Finetti P, Moreau T (2009). CD146 expression is associated with a poor prognosis in human breast tumors and with enhanced motility in breast cancer cell lines.. Breast Cancer Res.

[36] Grigoriu B, Chahine B, Zerimech F, Grégoire M, Balduyck M (2009). Serum mesothelin has a higher diagnostic utility than hyaluronic acid in malignant mesothelioma.. Clin Biochem.

[37] Shah CA, Lowe KA, Paley P, Wallace E, Anderson GL (2009). Influence of ovarian cancer risk status on the diagnostic performance of the serum biomarkers mesothelin, HE4, and CA125.. Cancer Epidemiol Biomarkers Prev.

[38] Wei Y, Hu G, Kang Y (2009). Metadherin as a link between metastasis and chemoresistance.. Cell Cycle.

[39] Weintraub SJ, Chow KN, Luo RX, Zhang SH, He S (1995). Mechanism of active transcriptional repression by the retinoblastoma protein.. Nature.

[40] Prockop DJ (1997). Marrow stromal cells as stem cells for nonhematopoietic tissues.. Science.

[41] Caplan AI, Dennis JE (2006). Mesenchymal stem cells as trophic mediators.. J Cell Biochem.

[42] Hynes RO, Lander AD (1992). Contact and adhesive specificities in the associations, migrations, and targeting of cells and axons.. Cell.

[43] Sellers WR, Novitch BG, Miyake S, Heith A, Otterson GA (1998). Stable binding to E2F is not required for the retinoblastoma protein to activate transcription, promote differentiation, and suppress tumor cell growth.. Genes Dev.

[44] Batsché E, Muchardt C, Behrens J, Hurst HC, Crémisi C (1998). RB and c-myc activate expression of the E-cadherin gene in epithelial cells through interaction with transcription factor AP-2.. Mol Cell Biol.

[45] Yang J, Mani SA, Weinberg RA (2006). Exploring a new twist on tumor metastasis.. Cancer Res.

[46] Arima Y, Inoue Y, Shibata T, Hayashi H, Nagano O (2008). Rb Depletion results in deregulation of E-cadherin and induction of cellular changes characteristic of the EMT.. Cancer Res.

[47] Polyak K, Weinberg RA (2009). Transitions between epithelial and mesenchymal states: acquisition of malignant and stem cell traits.. Nature Reviews Cancer.

[48] Kaye FJ (2002). RB and cyclin dependent kinase pathways: defining a distinction between RB and p16 loss in lung cancer.. Oncogene.

[49] Hahn WC, Weinberg RA (2002). Rules for making human tumor cells.. N Engl J Med.

[50] Chou MM, Blenis J (1996). The 70 kDa S6 kinase complexes with and is activated by the Rho family G proteins Cdc42 and Rac1.. Cell.

[51] Van Gelder RN, von Zastrow ME, Tool A, Dement WC, Barchas JD (1990). Amplified RNA synthesized from limited quantities of heterogeneous cDNA.. Proc Natl Acad Sci U S A.

[52] Lee EK, Park T (2007). Exploratory methods for checking quality of microarray data.. Bioinformation.

