# Supplementary figures and images for: A Role for the Retinoblastoma Protein As a Regulator of Mouse Osteoblast Cell Adhesion: Implications for Osteogenesis and Osteosarcoma Formation
Source: PLoS One. 2010 Nov 11;5(11):e13954. doi: 10.1371/journal.pone.0013954 (PMC2978706; doi:10.1371/journal.pone.0013954)

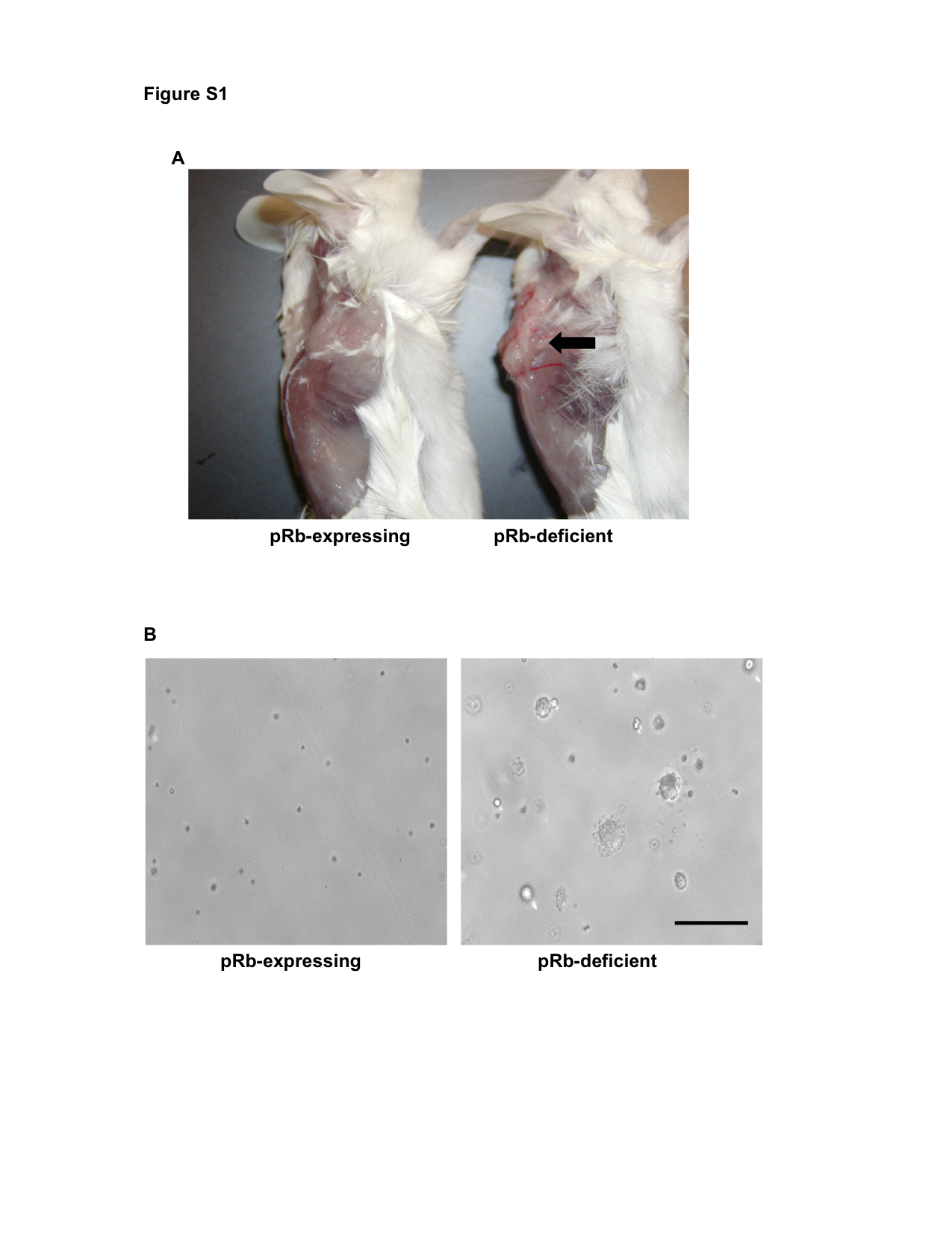

Supplement: Figure S1 — pRb-deficient osteoblasts show traits of the transformed phenotype. (a) To determine if pRb-deficient MC3T3 osteoblasts are able to form tumors in vivo, 1×10^6^ cells were injected subcutaneously into SCID/NCr BALB/C mice. Three weeks after injection, mice injected with pRb-deficient MC3T3 osteoblasts developed highly vascularized tumors (arrow), while no tumors were apparent in mice injected with pRb-expressing MC3T3 controls. (b) While pRb-expressing MC3T3 osteoblasts were unable to grow in soft agarose and remained as single cells (left), pRb-deficient MC3T3 osteoblasts were able to proliferate to form cell colonies in soft agar (right), indicating that they have the capacity to grow in an anchorage-independent manner. Magnification is 10X, bar = 20 µm. (6.32 MB TIF) [file pone.0013954.s001.tif]

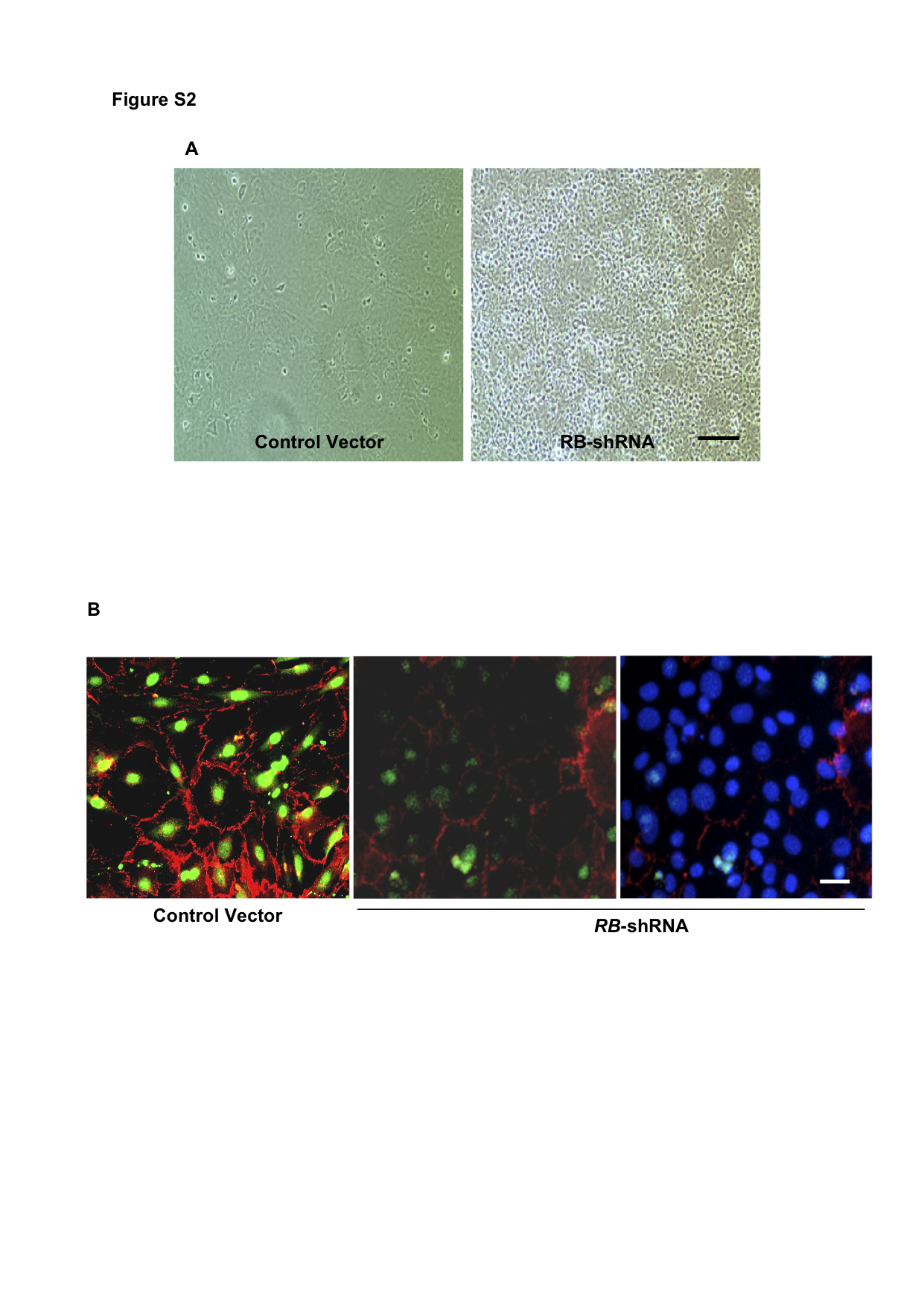

Supplement: Figure S2 — Knock-down of endogenous pRb in pRb-expressing osteoblasts disrupts adherens junctions. A DNA construct encoding a short hairpin-interfering RNA targeting the mouse RB gene (RB-shRNA) was cloned into an expression vector. pRb-expressing MC3T3 cells were then transfected with this construct followed by selection of stable transfectants. (a). Phase contrast micrographs at 4x showing that pRb-expressing MC3T3 in which pRb expression is suppressed by RB-shRNA (right) grow to a higher cell density when compared to pRb-expressing MC3T3 cells transfected with control vector (left). Bar = 2 µm. (b) Immunocytochemical localization of pRb and β-catenin in pRb-expressing MC3T3 osteoblasts transfected with control vector (left) showed nuclear immunoreactivity for pRb (green) and membrane-associated immunoreactivity for β-catenin (red). Immunocytochemical analysis of pRb-expressing MC3T3 cells transfected with the RB-shRNA (middle) showed a strong correlation between pRb and β-catenin expression in these cultures, pRb and β-catenin expression being undetectable in the same cells. Total nuclei stained with DAPI (blue) are shown in the right panel, magnification is 100×, bar = 1 µm. (6.53 MB TIF) [file pone.0013954.s002.tif]

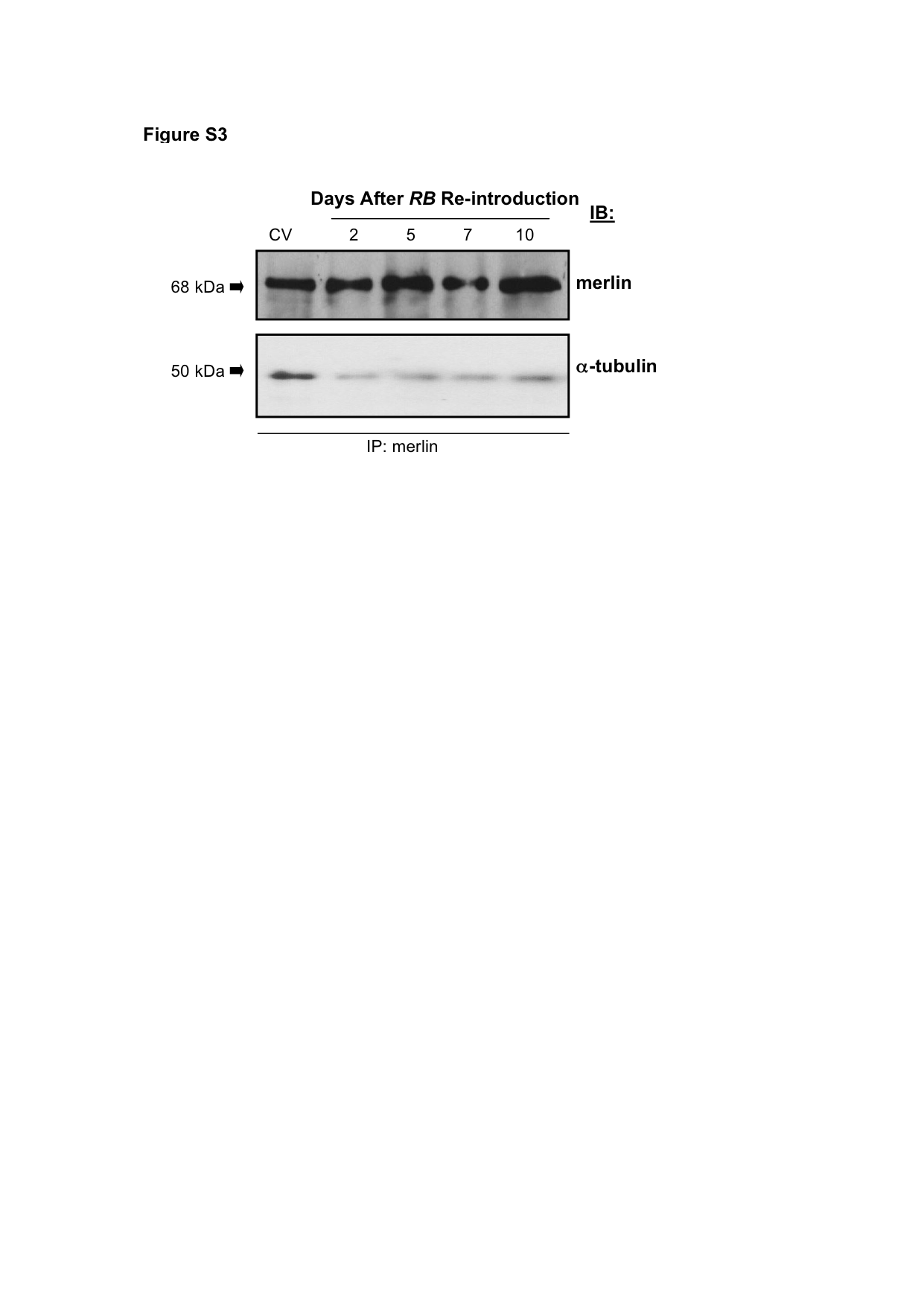

Supplement: Figure S3 — pRb regulates merlin folding in human osteoblasts. To determine if pRb expression is necessary and sufficient to promote merlin activation, we investigated merlin-tubulin interactions in the pRb-deficient osteosarcoma cell line Saos-2. Saos-2 cells were transfected with either control vector (CV) or with a vector transducing pRb. Cells transfected with control vector and pRb-transfected cells were cultured for 2, 5, 7, and 10 days following transfection and harvested to obtain whole protein lysates. Merlin was immunoprecipitated from these lysates using a merlin-specific antibody, followed by immunoblot analysis using antibodies against merlin and α-tubulin. Merlin is predominantly co-immunoprecipitated with α-tubulin in CV-transfected cells, while this interaction is significantly diminished as early as 2 days after pRb reintroduction, suggesting that merlin is activated soon after pRb reintroduction into Saos-2 cells. (6.53 MB TIF) [file pone.0013954.s003.tif]

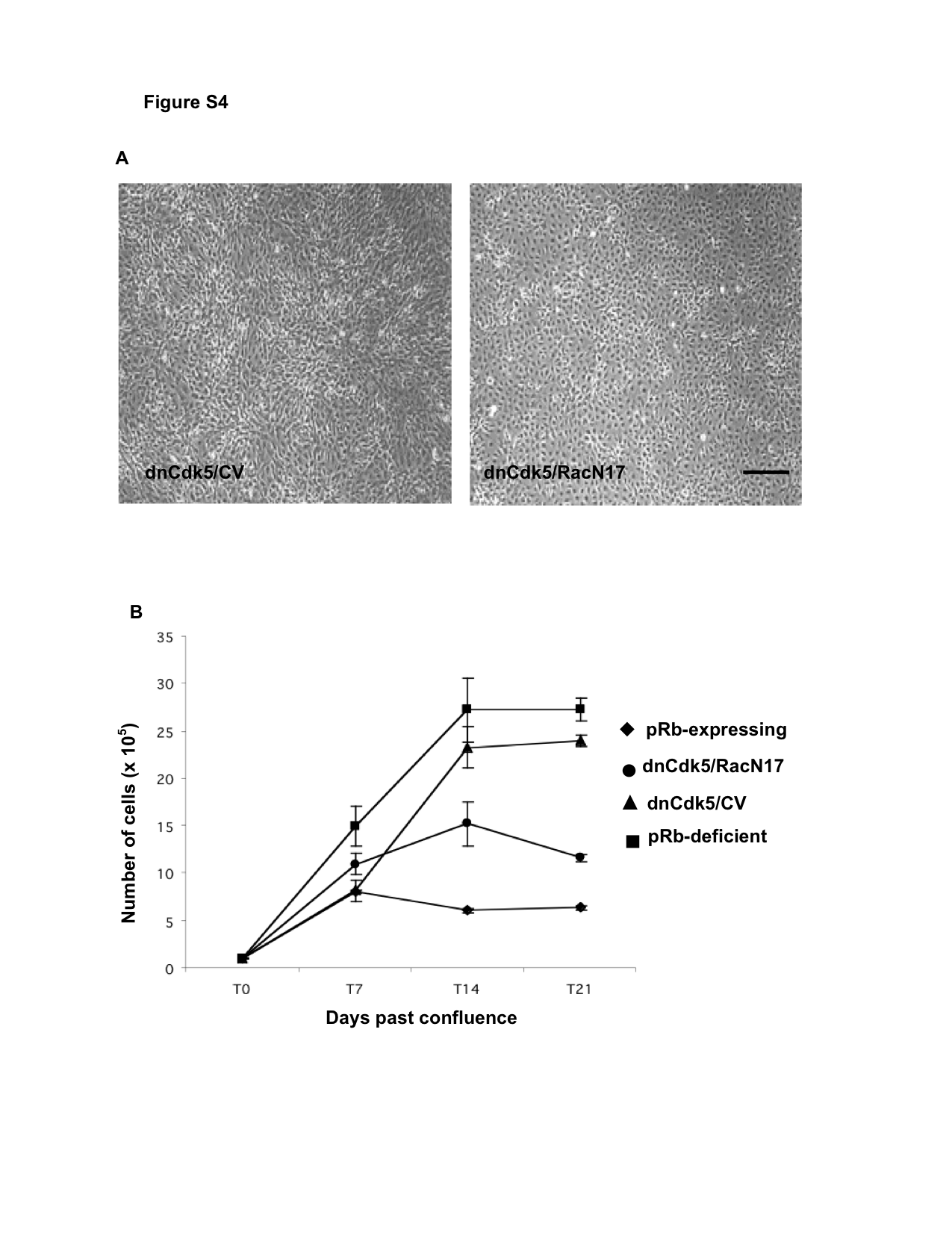

Supplement: Figure S4 — Blocking Rac1 activity reestablishes adherens junction formation and contact-dependent growth arrest. In these experiments, pRb-expressing osteoblasts stably expressing dnCdk5 were transfected with either a control vector or a dominant negative Rac1 (RacN17), followed by selection of stable transfectants. A–B. Phase contrast photomicrographs at 4× magnification (bar = 2 µm) (A) and growth curve analysis (B) showing that pRb-expressing osteoblasts stably expressing dnCdk5 and RacN17 grew in culture to a lower cell density than dnCdk5-expressing osteoblasts transfected with control vector (CV). In each graph each data point represents the mean of three independent experiments ± standard error. (c) Immunocytochemical localization of β-catenin showing that its membrane-associated localization is partially reestablished in pRb-expressing osteoblasts expressing both dnCdk5 and RacN17. Magnification is 100×, bar = 1 µm. (d) Crystal violet staining showing reduced number of foci in pRb-expressing osteoblasts expressing both dnCdk 5 and RacN17. (2.30 MB TIF) [file pone.0013954.s004.tif]
